# Examining technology-assisted rehabilitation for older adults’ functional mobility: a network meta-analysis on efficacy and acceptability

**DOI:** 10.1038/s41746-023-00907-7

**Published:** 2023-08-24

**Authors:** Błażej Cieślik, Justyna Mazurek, Adam Wrzeciono, Lorenza Maistrello, Joanna Szczepańska-Gieracha, Pierfranco Conte, Pawel Kiper

**Affiliations:** 1 Healthcare Innovation Technology Lab, IRCCS San Camillo Hospital, Venezia, 30126 Italy; 2 University Rehabilitation Centre, Wroclaw Medical University, Wroclaw, 50-367 Poland; 3 Faculty of Physiotherapy, Wroclaw University of Health and Sport Sciences, Wroclaw, 51-612 Poland; 4 IRCCS San Camillo Hospital, Venezia, 30126 Italy

**Keywords:** Geriatrics, Disability

## Abstract

Technological advancements facilitate feedback adaptation in rehabilitation through virtual reality (VR) exergaming, serious gaming, wearables, and telerehabilitation for older adults fall prevention. Although studies have evaluated these technologies, no comparisons of their effectiveness have been conducted to date. Thus, this study aims to assess the differences in effectiveness of these interventions on balance and functional mobility in the older adults. A systematic review and network meta-analysis (NMA) were conducted to identify the most effective interventions for improving balance and functional mobility in adults aged 60 and over. The search was conducted in five databases (PubMed, Embase, Cochrane Central Register of Controlled Trials, Scopus, and Web of Science) up to June 10, 2023. The eligibility criteria were: (1) older adults, (2) functional mobility, balance, or gait as the primary outcome, (3) new technology intervention, and (4) randomized study design. New technology interventions were classified into five categories: exergaming with balance platforms or motion capture technologies, other serious gaming, interventions with wearables, and telerehabilitation. Additionally, two categories of control interventions (conventional exercises and no treatment) were extracted. The NMA was performed for the aggregated results of all outcomes, and separately for clinical functional scales, functional mobility, and gait speed results. Fifty-two RCTs with 3081 participants were included. Exergaming with motion capture was found to be statistically significant in producing a better effect than no treatment in the analysis of the functional mobility with an SMD of −0.70 (*P* < 0.01). The network meta-analysis revealed that exergaming with motion capture offers greater therapeutic benefits for functional mobility and balance compared to no treatment control. The effectiveness of this approach is similar to that of conventional exercises. Further RCTs are needed to provide a more definitive conclusion, particularly with respect to the effectiveness of serious games, telerehabilitation, and interventions with wearables.

## Introduction

According to the projections of the United Nations World Population Prospects, by 2050, one in four people living in Europe and Northern America will be 65 years or older^1^. Among the numerous challenges associated with an aging population are those related to falls and the resultant consequences. The World Health Organization reports that every year, between 28 and 35% of seniors aged 65 and above fall, and this rate increases to between 32 and 42% for individuals over 70 years old^2^. Data indicate that falls are the leading cause of both fatal and non-fatal unintentional injuries for this group of people^3^.

Accordingly, extensive studies have been conducted to discover effective forms of balance enhancement, thereby reducing the risk of falls. Approaches such as exercise, assistive technology, examination and adjustments to the environment, and the implementation of quality improvement strategies, have all been demonstrated to be valuable components of fall prevention programs^4^. A supplementary mechanism that could be utilized in balance training is feedback. It is a form of sensory augmentation in which information regarding the output or result of a system is utilized to modify or regulate the input or future behavior of that same system. In medical research, augmented feedback is used to induce enduring changes in motor learning and attain superior performance^5^. For balance training, augmented feedback is most commonly provided in the form of visual displays, tactile and kinesthetic perception stimuli, or a combination of these modalities^6,7^.

Technological advancements in wearables and virtual reality (VR) have facilitated the integration of feedback in rehabilitation medicine. Multiple studies have been conducted to investigate the efficacy of combining various forms of VR with feedback to enhance postural stability and balance^8^. For instance, visual feedback has been utilized in various force plates used in exergaming. These devices transfer the center of pressure (COP) record to the screen in real-time, allowing the patient to control and adjust the COP’s displacement^9^. Another mechanism frequently utilized in exergaming is motion capture. It is a method used to capture the movements of the participant and convert them into a digital (on screen) representation of the motion, thus providing visual feedback and enabling real-time correction of the exercises performed^10^. The advantage of exergaming is the accessibility of equipment and commercially available game software; however, its effectiveness may be limited as it is not specifically designed for rehabilitation purposes. Hence, researchers are developing specialized software called ‘serious games’ that target special interest groups by combining the desired and measurable outcomes (serious aspect), in-game functional messaging, skills learning and entertainment (game aspect) simultaneously^11^. Telerehabilitation, which has recently gained wider recognition, can also provide visual feedback, particularly through the use of inertial sensors^12^. These sensors, when employed as wearable technology, can also provide haptic feedback that can be used for posture adjustments^13^.

The efficacy of the previously mentioned mechanisms for balance training has been separately evaluated in numerous systematic reviews and meta-analyses; however, no study has directly compared their effectiveness to each other^14–19^. Therefore, the main aim of this study was to quantitatively evaluate the differences in the effectiveness of virtual reality exergaming, serious gaming, interventions with wearables, and telerehabilitation compared to conventional treatments or no treatment on balance and functional mobility in the older adults. Additionally, this study aimed to assess the acceptability of interventions based on new technologies for functional rehabilitation.

Our study’s key findings reveal that VR exergaming incorporating motion capture technology yields substantial improvements in functional mobility and balance among older adults, similar to conventional exercises. This intervention proves more effective than exergaming with balance boards, serious games, wearables, and telerehabilitation. When assessing dropout rates, new technologies demonstrated comparable patient acceptability to the control group, although the pooled results indicated control interventions exhibited a statistically significant 2-percentage-point lower rate.

## Results

**Fig. 1:**
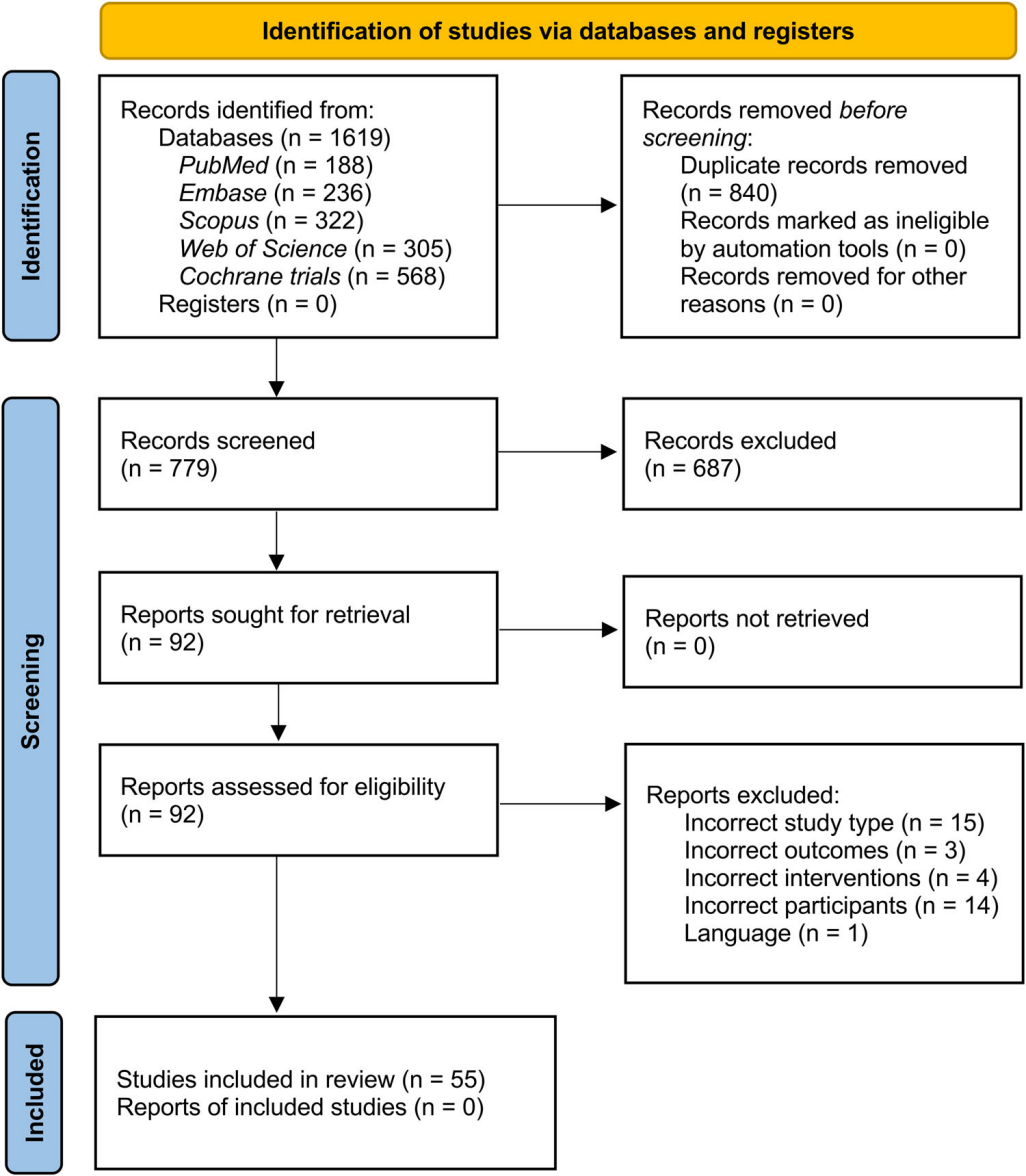
Study flow diagram.

The flow of the study’s identification and selection process is shown in Fig. 1. The systematic search yielded 1619 records, of which 779 remained after removing duplicates and screening titles and abstracts. Following the initial screening procedure, 92 articles were considered for the full-text review. After a full-text assessment, 55 RCTs involving 3273 enrolled participants were included in the NMA (Fig. 1).

### Characteristics of the included studies

Among the included studies, exergaming with balance platforms^20–41^ (*n* = 22, total participants: 1084) and motion capture^42–58^ (*n* = 17, total participants: 912) were the most frequently investigated interventions. Seven studies (total participants: 452) examined the use of other serious games^59–65^, four studies (total participants: 98) examined interventions with wearables^66–69^, and three studies (participants: 619) investigated telerehabilitation^70–72^. Two studies could not be classified into either group as it involved an intervention with the Nintendo Switch console^73,74^.

The sample size of the individual trials ranged from 12 to 503 participants, with a mean sample size of 58. All studies reported the mean ages of participants, which ranged from 63.3 to 86.2 (mean age: 73.7 years old). The total amount of therapy varied among the studies, ranging from 6 to 52 sessions (mean 23.6) of practice spread over a mean training period of 8.1 weeks (range 2–104 weeks), with a mean session duration of 48.9 min (range 15–60 min). The average sample size for exergaming with a balance board was 48.9 participants with a mean age of 74.8 years. Participants attended an average of 18.9 sessions, spread over 7.9 weeks, each lasting 39.6 min on average. For exergaming with motion capture, the mean sample size was 54 participants (mean age: 71.2), the mean number of sessions was 23.8, the mean number of weeks was 7.6, and the mean session length was 43 min. The mean sample size for other serious games interventions was 65 participants (mean age: 75.5) with an average of 20.9 sessions conducted over 8.1 weeks and lasted 41 min each. For interventions with wearables, the average sample size was 24 participants with a mean age of 77.4, who participated in 26 sessions over 7.6 weeks with an average session length of 43 min. For telerehabilitation, the mean sample size was 206 participants (mean age: 70.7), the mean number of sessions was 34.5, the mean number of weeks was 42.3 and the mean session length was 40 min. The study characteristics are summarized in Supplementary Table 1.

### Assumption assessment results

The studies included in Network 1 (All Outcomes) showed considerable heterogeneity, with a value of I^2^ = 49.6% (CI 33.6%; 60.8%). The heterogeneity test conducted for Network 2 (Functional Scales) and Network 3 (TUG) in the NMA resulted in I^2^ values of 56.6% (CI 26.5%; 74.4%) and 68.9% (CI 53.1%; 79.4%), respectively. However, for Network 4 (Gait Speed), the results did not support consistency, as indicated by a high heterogeneity of I^2^ = 97.7% (CI 96.5%; 98.5%). Since the results did not meet the NMA assumptions, they were not presented here. Nevertheless, they are included as Supplementary Information, along with a detailed description of the assumptions assessment results (Supplementary Figs. 2, 3).

### NMA results and ranking of the treatments

**Fig. 2:**
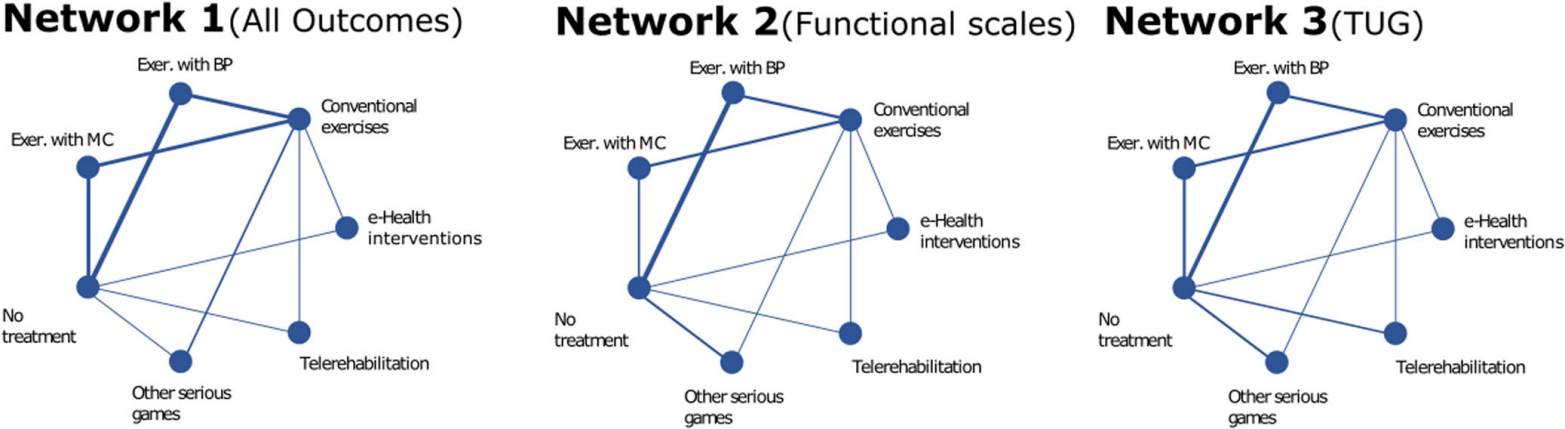
Network plot presenting the trial data contributing evidence comparing exercise treatment types for each network. The size of the nodes represents how many times the exercise appears in any comparison about that treatment and the width of the edges represents the total sample size in the comparisons it connects.

**Fig. 3:**
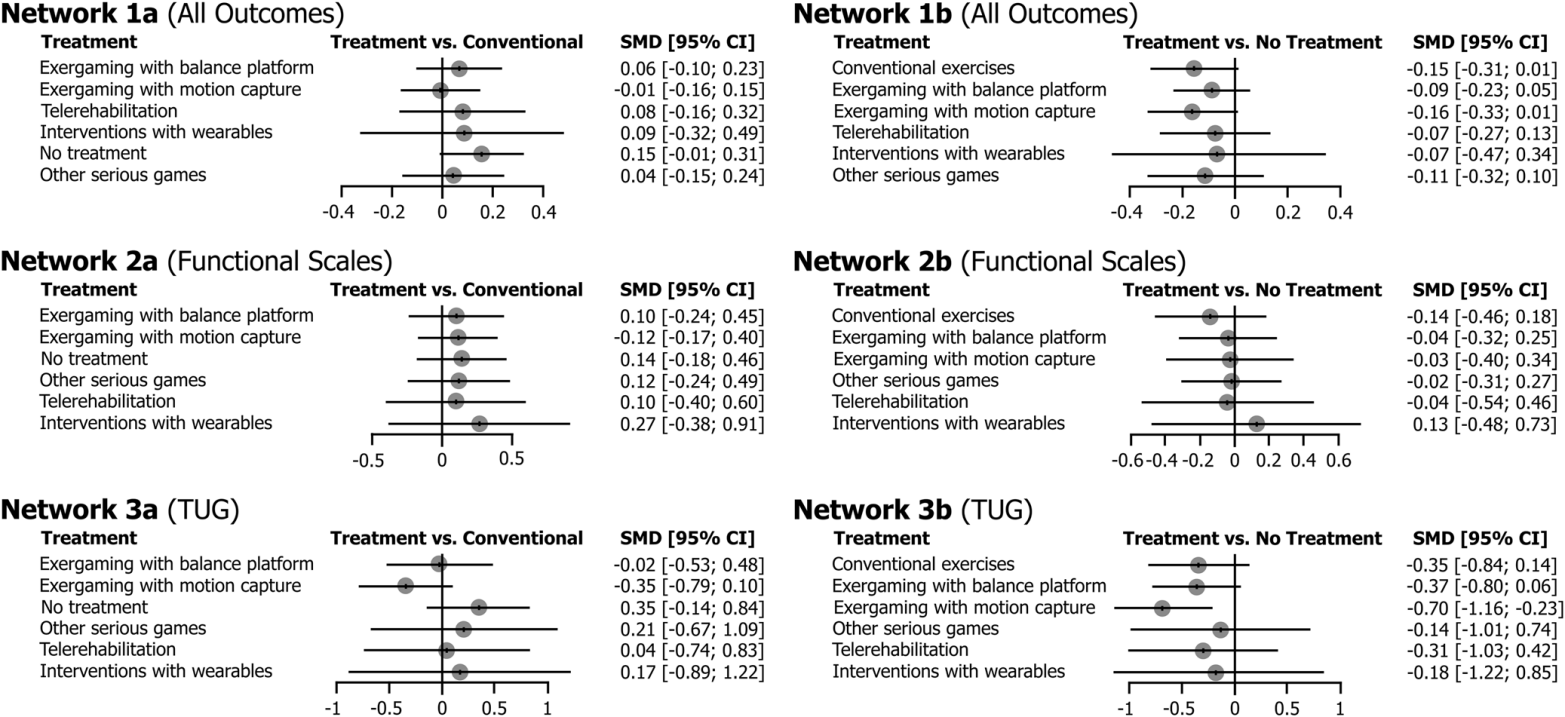
Summary network meta-analysis results for each intervention compared with conventional exercises (1a, 2a, and 3a) and no treatment (1b, 2b, and 3b). The circles on the graph represent the estimate of the effect for each study, while the horizontal lines that intersect the circles represent their 95% CI.

Figure 2 illustrates the network plot for each of the selected network analyses. The main result of the NMA was that exergaming with motion capture was found to be on the verge of statistical significance in producing a better effect than no treatment in Network 1 (All Outcomes), with a SMD of −0.16 (CI −0.33; 0.01, *P* = 0.06), and statistically significant in Network 3 (TUG), with a SMD of −0.70 (CI −1.16; −0.23, *P* < 0.01) (Table 1). However, the interventions did not yield significantly better response rates than conventional exercises in each of the selected networks, with 95% confidence intervals not crossing the null (Fig. 3). Supplementary Table 2 contains total direct and indirect effect estimates.

**Table 1:** Estimated effect size results.

Intervention	Studies (*n*)	Participants (*n*)	Effect size (95% CI), *P* value		*P*-score
			Compared to CE	Compared to NT	
Network 1 (All Outcomes)					
Exer. with MC	11	440	0.01 [−0.15; 0.16], *P* = 0.94	−0.16 [−0.33; 0.01], *P* = 0.06	0.72
Other serious games	3	150	−0.04 [−0.24; 0.15], *P* = 0.67	0.11 [−0.10; 0.32], *P* = 0.31	0.55
Exer. with BP	16	706	−0.06 [−0.23; 0.10], *P* = 0.47	−0.09 [−0.23; 0.05], *P* = 0.22	0.48
Interv. with wearables	3	73	−0.09 [−0.49; 0.32], *P* = 0.68	0.07 [−0.34; 0.47], *P* = 0.75	0.45
Telerehabilitation	2	482	−0.08 [−0.32; 0.16], *P* = 0.50	0.07 [−0.13; 0.27], *P* = 0.48	0.43
Network 2 (Functional Scales)					
Exer. with BP	5	147	−0.10 [−0.45; 0.24], *P* = 0.55	−0.04 [−0.32; 0.25], *P* = 0.92	0.52
Telerehabilitation	1	34	−0.12 [−0.49; 0.24], *P* = 0.69	0.04 [−0.46; 0.54], *P* = 0.87	0.52
Other serious games	3	203	−0.14 [−0.46; 0.18], *P* = 0.50	0.02 [−0.27; 0.31], *P* = 0.90	0.48
Exer. with MC	4	168	−0.12 [−0.40; 0.17], *P* = 0.42	−0.03 [−0.40; 0.34], *P* = 0.98	0.48
Interv. with wearables	2	43	−0.10 [−0.60; 0.40], *P* = 0.41	−0.13 [−0.73; 0.48], *P* = 0.68	0.31
Network 3 (TUG)					
Exer. with MC	9	354	0.35 [−0.10; 0.79], *P* = 0.13	−0.70 [−1.16; −0.23], *P* < 0.01	0.89
Exer. with BP	9	351	0.02 [−0.48; 0.53], *P* = 0.93	−0.37 [−0.80; 0.06], *P* = 0.09	0.58
Telerehabilitation	2	241	−0.04 [−0.83; 0.74], *P* = 0.91	0.31 [−0.42; 1.03], *P* = 0.41	0.51
Other serious games	2	181	−0.21 [−1.09; 0.67], *P* = 0.64	0.14 [−0.74; 1.01], *P* = 0.76	0.37
Interv. with wearables	2	42	−0.17 [−1.22; 0.89], *P* = 0.75	0.18 [−0.85; 1.22], *P* = 0.73	0.18

In terms of ranking, Network 1 (All Outcomes) found that exergaming with motion capture (*P*-score of 72%) had a similar probability to produce therapeutic benefits as conventional exercises (71%) (Table 1). Other serious games (55%), exergaming with a balance platform (48%), interventions with wearables (45%), and telerehabilitation (43%) were found to be better treatments in the network than no treatment (16%). In Network 2 (functional scales), the *P*-score obtained by each intervention was similar to no treatment (43%), and below conventional exercises (75%). In Network 3 (TUG), exergaming with motion capture (89%) and balance platform (58%) had *P*-scores above conventional exercises (55%). On the other hand, other serious games, telerehabilitation, and interventions with wearables had *P*-scores similar or lower than no treatment (42%, 37%, and 18%, respectively).

### Acceptability: drop-out analysis

A total of 51 trials, derived from 45 studies, were included in the proportional drop-out meta-analysis. When considering all pooled treatments among 2613 participants, 324 dropouts were reported, resulting in a prevalence of 12.4% (CI 8.4%; 13.4%). The dropout rate for technology-based interventions was 13.5% (CI 11.1%; 15.8%), compared to 11.3% (CI 9.1%; 14.1%) for the CE control group. The main findings indicated a significant difference, resulting in slightly higher probability of drop-out in technology-based interventions than in the CE group (OR = 1.22; CI 1.03; 1.45; *P* = 0.03) (Supplementary Fig. 4). No significant heterogeneity was found between the studies (I^2^ = 0.0%; CI 0.0%; 79.2%; *P* = 0.90). There were no statistically significant differences when comparing drop-out rate within technology-based interventions.

### Risk of bias assessment

**Fig. 4:**
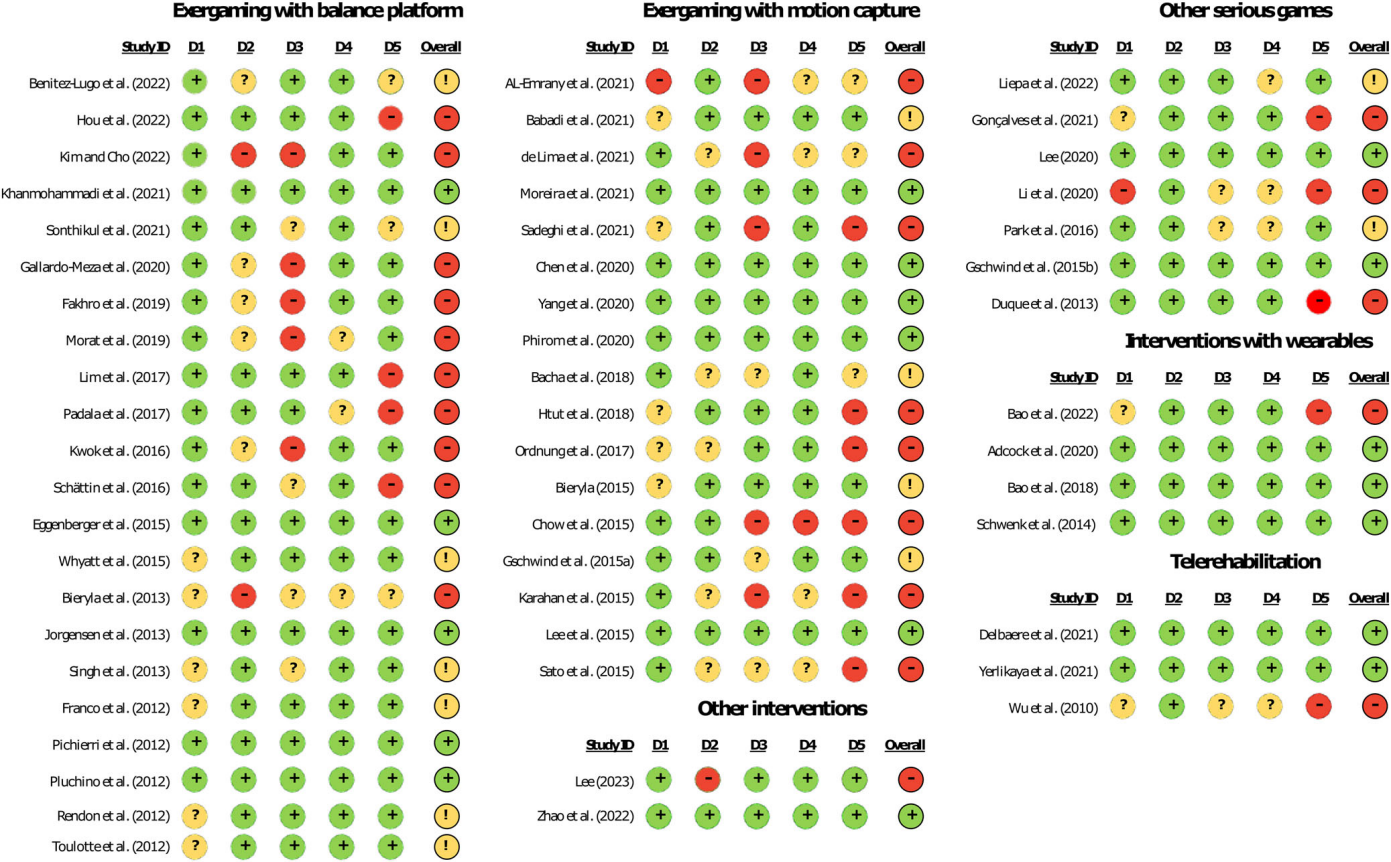
Risk of bias assessment results. (+) Low risk; (−) High risk; (?) Some concerns; D1: Randomization process; D2: Deviations from the intended interventions; D3: Missing outcome data; D4: Measurement of the outcome; D5: Selection of the reported results.

Figure 4 illustrates the results of the risk of bias assessment. Thirty-two percent (18 out of 55), 24% (13 out of 55), and 44% (24 out of 55) of the studies had a low, some concerns, and high overall risk of bias, respectively. One in three studies had some concerns about the risk of bias for allocation concealment (i.e., concealment method not described or insufficient detail to allow judgment). Furthermore, 21 studies (38%) had some concerns or a high risk of bias for selective outcome reporting, mainly due to incomplete or missing data necessary to conduct the NMA. Considering the analysis of the interventions, the level of risk of bias was found to be similar across them. In the studies included, exergaming with a balance platform had a high risk of bias of 45%, motion capture had 47%, other serious games had 42%, interventions with wearables had 25%, and telerehabilitation had 33%.

## Discussion

The primary objective of this study was to conduct a quantitative evaluation of the disparities in effectiveness between new technology interventions and conventional treatments, or no treatment at all, with regard to balance and functional mobility in the older adults. The main finding of this study was that exergaming with motion capture was found to be significantly more effective than no treatment in improving balance and functional mobility in the older adults. Additionally, in terms of ranking, exergaming with motion capture was comparable to conventional exercises, particularly in the context of Network 1 (All Outcomes), and superior to conventional exercise in Network 3 (TUG). However, no statistically significant differences were observed between the other interventions and either conventional exercise or no treatment.

The superiority of exergaming with motion capture over other new technology interventions can be attributed to its ability to provide comprehensive feedback to the patient. This intervention entails a visual representation of the patient’s entire body on the screen, thereby facilitating greater mobility that more closely reflects activities performed during balance functional scales and functional mobility tests. Conversely, exergaming with a balance platform relies on feedback using the COP, which restricts mobility to the patient’s stability limits. As a consequence, this approach may hinder the transfer of motor skills acquired during training to functional test results. Given that augmented feedback is a crucial element of motor learning, a more comprehensive feedback mechanism has the potential to enhance the translation of these skills into clinical functioning^5^. A meta-analysis from 2020 produced comparable outcomes to no treatment, however, the analysis was conducted on exergaming as a collective whole and did not specify the effectiveness of individual technologies^75^. In 2016, Donath and colleagues conducted a study comparing exergaming to conventional balance training, and obtained outcomes similar to ours, ultimately concluding that traditional exercise is marginally superior^76^. A recent meta-analysis examining both conventional exercises and no-exercise as a control interventions concluded that exergaming has the potential to improve specific physical function domains in older adults^77^. However, none of the previously mentioned meta-analyses differentiated motion capture and balance platform as separate forms of exergaming.

It is noteworthy that serious games, which are software programs explicitly designed for rehabilitation purposes, exhibit lower effectiveness than anticipated. Considering their purposeful design and intended functions, it would be reasonable to expect a higher degree of efficacy. Although meta-analyses have investigated the effectiveness of serious games in enhancing cognitive functions among older adults, no such analysis has assessed their efficacy in improving balance and functional mobility in the older adults^78^. This research gap could potentially be attributed to the primary application of serious games in treating neurological diseases^79–81^. Similarly, in our study, interventions involving wearables did not demonstrate significantly higher effectiveness in augmenting balance. This outcome may be related to the fact that such devices often employ custom-made software, which exhibit considerable diversity in their approaches, consequently leading to inconsistent results. In a recent systematic review on the application of eHealth interventions in balance treatment, Gaspar and Lapão (2020) reported that the employed methodologies did not allow for a definitive comparison of the results, and they recommended more rigorous investigations^16^. The aforementioned findings regarding serious games and wearable interventions should be interpreted within the context of their costs. A recent study examining the costs of an off-the-shelf exergame intervention in patients with heart failure demonstrated relatively low costs, and it was observed that patients were willing to cover more than half of the intervention expenses^82^. In contrast, the development and ongoing software updates of a serious game program can be financially demanding and resource-intensive, involving multiple stakeholders such as experts, game developers, and software engineers^83,84^.

Furthermore, when comparing the drop-out rate within new technologies, we provided evidence that each of them is equally acceptable to patients as the control group. However, considering the pooled acceptability results, the control interventions exhibited a statistically significant 2-percentage-point lower drop-out rate than the new technology interventions. This difference can be attributed to the fact that advanced age can influence the acceptability of using new technologies^85^. Additionally, Chen et al. (2018) demonstrated that perceived playfulness and perceived usefulness are two primary factors influencing seniors’ willingness to engage in exergames^86^. In light of the obtained results, the latter factor appears to be particularly significant because, compared to conventional professional-guided exercises, commercial games may not be perceived as equally useful as standard exercises. These results differ slightly from a 2018 systematic review that concluded that technology-based exercise interventions have similar adherence to traditional exercise programs^87^. However, it is worth noting that the review employed a qualitative analysis method and included less than half the number of articles compared to this NMA.

Exergaming, particularly the type that incorporates motion capture technology, can offer patients an accessible way to engage in feedback-based exercises without requiring professional supervision. The benefits of accessibility render exergaming a viable intervention for enhancing balance and functional mobility among older adults. Therefore, it is reasonable to consider the deployment of this type of intervention as either a standalone treatment or as a complementary measure alongside traditional rehabilitation programs. In addition to its clinical effectiveness, exergaming has been found to be a safe form of exercise, exhibiting minimal to no adverse effects^88^. The confluence of this study results, coupled with its safety and potential efficacy in enhancing the cognitive capabilities of older adults, positions exergaming with motion capture technology as a compelling therapeutic tool that may prove valuable in nursing home settings or for individual use by older persons in their homes. Serious games offer a primary advantage of greater individualization, making them particularly suitable for personalized medicine. Similarly, interventions involving wearables provide the same advantage, along with enhanced convenience and accessibility. Conversely, telerehabilitation holds a distinct advantage in its ability to deliver services remotely. This characteristic allows for increased access to specialized rehabilitation expertise and ensures continuity of care, particularly for individuals residing in remote or underserved areas.

A crucial factor in evaluating the effectiveness of an intervention is its dose, which includes elements such as frequency, intensity, duration, and timing^89^. However, the number of intervention sessions conducted varied significantly among the articles analyzed in this study. This difference is important since the number of interventions can impact the therapy’s effectiveness and, consequently, the results of the meta-analysis. To enhance the comparability of studies, future research should focus on identifying the appropriate number of sessions, their duration, and the weeks of intervention. This is particularly important for interventions that are self-directed and conducted by the patient at home without therapist supervision. Given the substantial variability and customization of software utilized in serious games, wearables interventions, and telerehabilitation, it is imperative to evaluate the cost-effectiveness of these interventions, especially when compared to commercially available exergames. However, more studies evaluating the efficacy of these interventions are needed as the current body of research is limited. Furthermore, most of the studies conducted so far have employed clinical functional scales, and it would be valuable to enhance future research efforts by incorporating measurements of COP and conducting 3D gait analyses. Another area worth exploring is the comprehensive implementation of the gamification process, which involves incorporating competitive elements like points or leaderboards. Senior users, particularly in the health domain, could potentially benefit from gamification, and a full adoption of this approach may lead to increased engagement in interventions and a reduction in the dropout rate^90^. Finally, the use of the Nintendo Switch in the field of exergaming is worth exploring. Although there has been little research done on this topic so far, the device offers additional accessories that enable feedback during resistance, aerobic, and balance exercises^91^.

There are several limitations to consider when interpreting the obtained results. One major limitation is the small number of studies included in some interventions, such as wearables (4 studies) and telerehabilitation (3 studies), as well as the presence of small sample sizes in some studies, which may have resulted in less robust quantitative findings. Additionally, the RCTs had varying intervention durations, ranging from 6 to 52 sessions, and the studies in the current NMA had relatively short follow-up durations, with an average follow-up of 8 weeks (excluding telerehabilitation). Moreover, except for telerehabilitation, the average number of participants per study was low, ranging from 24 to 65. The interpretation of the results is significantly constrained due to the presence of risk of bias. Only 32% of the included studies received a low risk-of-bias assessment. Specifically, when considering exergaming with motion capture, the proportion of studies categorized as low risk was 29%, while a considerable 47% were classified as high risk. Another limitation is the combining of all types of control exercises into a single category of “conventional exercises” and interventions without exercises into a single category of “no treatment”, which prevented quantification of the comparative effectiveness of specific interventions within the same category. In Network 4 (Gait Speed) analysis, the data failed to meet the assumptions of statistical analysis due to a high degree of heterogeneity among the included studies, rendering the results inconclusive. Lastly, a significant number of studies reported results as median and IQR, which had to be transformed into mean and SD for use in NMA. Although this transformation is commonly used, it can potentially distort the results obtained.

Based on the results of this network meta-analysis, exergaming incorporating motion capture technology may provide therapeutic benefits for functional mobility and balance compared to no treatment control, exhibiting comparable effectiveness to conventional exercises. However, the interpretation of the results is significantly constrained by the presence of risk of bias, with only 32% of the included studies receiving a low risk-of-bias assessment. Additional high quality RCTs are required to draw a more definitive conclusion, particularly concerning the effectiveness of other serious games, telerehabilitation, and interventions involving wearables. This could lead to the identification of quantitative and objective tools to aid balance treatment in fall prevention programs. It is also crucial in terms of supplementing these programs with interventions that patients can perform at home without therapist supervision, potentially improving functional mobility and reducing the risk of falls.

## Methods

### Design

In order to jointly estimate the relative effectiveness of different treatments provided to patient, this study was designed as a systematic review with network meta-analysis (NMA). We combined direct evidence, i.e., that directly observable from the selected studies, and indirect evidence, i.e., that obtained through one or more common comparators^92^. The study followed the Preferred Reporting Items for Systematic Reviews and Meta-analyses 2020 and AMSTAR2 guidelines^93,94^. The review protocol was registered a priori in the PROSPERO database (CRD42022376092).

### Literature search and study selection

MEDLINE (via PubMed), Embase, Cochrane Central Register of Controlled Trials, Scopus, and Web of Science databases were searched up to June 10, 2023. Please see the Supplementary Information for full search strategy used in the databases.

Studies identified from search databases were assessed by two independent authors (JM and AW) according to the following eligibility criteria: (1) targeted participants were limited to healthy older adults (aged 60 years or older); (2) functional mobility, balance, or gait specified as the primary outcome measure; (3) intervention defined as virtual reality exergaming, other serious gaming, interventions with wearables, or telerehabilitation, with at least one control group in the trial (eligible comparators included no treatment, usual care, conventional rehabilitation, exercises, or unrelated interventions); and (4) studies with a randomized design. Studies were excluded if the targeted participants were restricted to specialized populations (with neurodegenerative diseases, neurological, orthopedic, or pulmonary disorders, and cancers). Studies were also excluded if the intervention included robotic or exoskeleton devices.

The primary outcomes were changes for functional mobility, balance in the scores of the rating scales and for gait in gait speed after the interventions. Outcomes were measured using the Timed Up and Go test (TUG), Center of Pressure (COP), Berg Balance Scale (BBS), Short Physical Performance Battery (SPPB), Five-Time Sit to Stand Test (5XSST), Functional Reach Test (FRT), Single Leg Stance (SLS), Fullerton Advanced Balance Scale (FAB), Overall Stability Index (OSI), Tinetti Performance Oriented Mobility Assessment (POMA), and Mini-Balance Evaluation Systems Test (Mini-BESTest). The secondary outcome was acceptability, measured as the dropout rate (defined as leaving the study before its end for any reason).

The studies retrieved by the search strategy, along with study information and abstract text, were imported into the Systematic Review Assistant-Deduplication Module for de-duplication^95^. Articles were divided between two reviewers (JM and AW), with each title and abstract independently screened using Rayyan AI software^96^. After the initial literature search was conducted, BC and PK retrieved and independently screened the full-text articles. Conflicts over inclusion were resolved through discussions. Data were extracted by a single reviewer (BC) and checked by JSzG.

### Data extraction

From each study, we extracted the following information: the number of participants included (along with the average age), the number of sessions of the intervention received and the length of a single session, the outcome measures used, the number of dropouts in the groups, and the study conclusion.

New technology interventions were classified into 5 categories: (1) exergaming with balance platform, including all kinds of devices which could provide feedback though stepping on it; (2) virtual reality exergaming with motion capture, defined as a device which could provide feedback via recording the movement; (3) other serious gaming, including all kinds of devices and software’s created for rehabilitation purposes; (4) interventions with wearables, including mobile phones and wearables; (5) telerehabilitation (including all forms of remote support aimed at balance and functional mobility training). Additionally, we extracted two categories of control interventions: (6) conventional rehabilitation, including all forms of traditional rehabilitation, physical exercises, and treatment as usual; and (7) no treatment (defined as no intervention or interventions without physical exercises, such as education and cognitive training).

### Quality assessment

Two authors (JM and AW) independently assessed the quality of the included studies using version 2 of the Cochrane risk of bias tool for randomized trials (RoB2), with any disagreements resolved by a third researcher (BC)^97^. The bias risk assessment included seven criteria: random sequence generation (selection bias), allocation concealment (selection bias), blinding of participants and personnel (performance bias), blinding of outcome assessment (detection bias), incomplete outcome data (attrition bias), selective reporting (reporting bias), and other biases.

### Data analysis

The analyses were carried out with R Studio v4.2.2 software with the *netmeta* package for frequentist analysis. For each study that presented pre- and post-treatment mean and standard deviation values, the Standardized Mean Difference (SMD) and Standard Error (SE) were calculated both within and between treatment groups. When the studies provided median values and quartiles of the distribution, the SMD and SE values were calculated, first calculating the pre- and post-treatment mean and standard deviation values for each group. The frequentist weighted least squares approach was applied to the network meta-analysis with random model. For each treatment comparison, the direct and indirect effect, associated with the 95% confidence interval (95% CI), were estimated. The data analysis was performed for four outcome sets. Network 1 (All Outcomes) represents the aggregated results of all balance related outcomes (COP, BBS, SPPB, FAB, POMA, Mini-BESTest, 5xSST, FRT, SLS, and TUG), Network 2 (Functional Scales) comprises the clinical functional scales, including BBS, SPPB, FAB, POMA, and Mini-BESTest. Network 3 (TUG) encompasses the functional mobility results based on the TUG test, while Network 4 (Gait Speed) includes the results of quantitative gait speed analysis. Heterogeneity between studies was assessed using generalized Cochran’s Q statistic (Supplementary Fig. 2). In addition, the I-square inconsistency index (I^2^) was used to quantify the percentage of variability among studies due to heterogeneity rather than chance. Heterogeneity was calculated both within (Qw) and between (Qb) studies. A significant Q value (*P*_Q_ < 0.05) indicates a lack of homogeneity of results among studies. The hypothesis of consistency between designs was tested by performing a generalized between-designs Cochran’s Q statistic. The significance of the difference between effect estimates based on direct and indirect evidence (*P* < 0.05) was an indication of significant disagreement (inconsistency). The change of the inconsistency contribution of single designs has been investigated in more detail by the Net Heat plot and Net splitting method. Finally, the comparative advantages of the treatments were investigated, calculating *P*-score values, i.e., the cumulative probability that treatments with the highest priority and those with the lowest priority are selected. To test whether the probability of dropout was higher in the treatment or control group, a binary meta-analysis based on odds ratios (OR) was conducted. The statistical significance was set at *α* < 0.05.

### Reporting summary

Further information on research design is available in the Nature Research Reporting Summary linked to this article.

### Supplementary information

Supplementary Information

Reporting Summary

## Data Availability

The search strategy is available in the Supplementary Information, and any additional data are available on reasonable request to the corresponding author.
