# Supplementary material for: Examining technology-assisted rehabilitation for older adults’ functional mobility: a network meta-analysis on efficacy and acceptability
Source: NPJ Digit Med. 2023 Aug 24;6:159. doi: 10.1038/s41746-023-00907-7 (PMC10449892; doi:10.1038/s41746-023-00907-7)
Supplement: Supplementary file 1 — Supplementary Information [file 41746_2023_907_MOESM1_ESM.pdf]

Supplementary Information

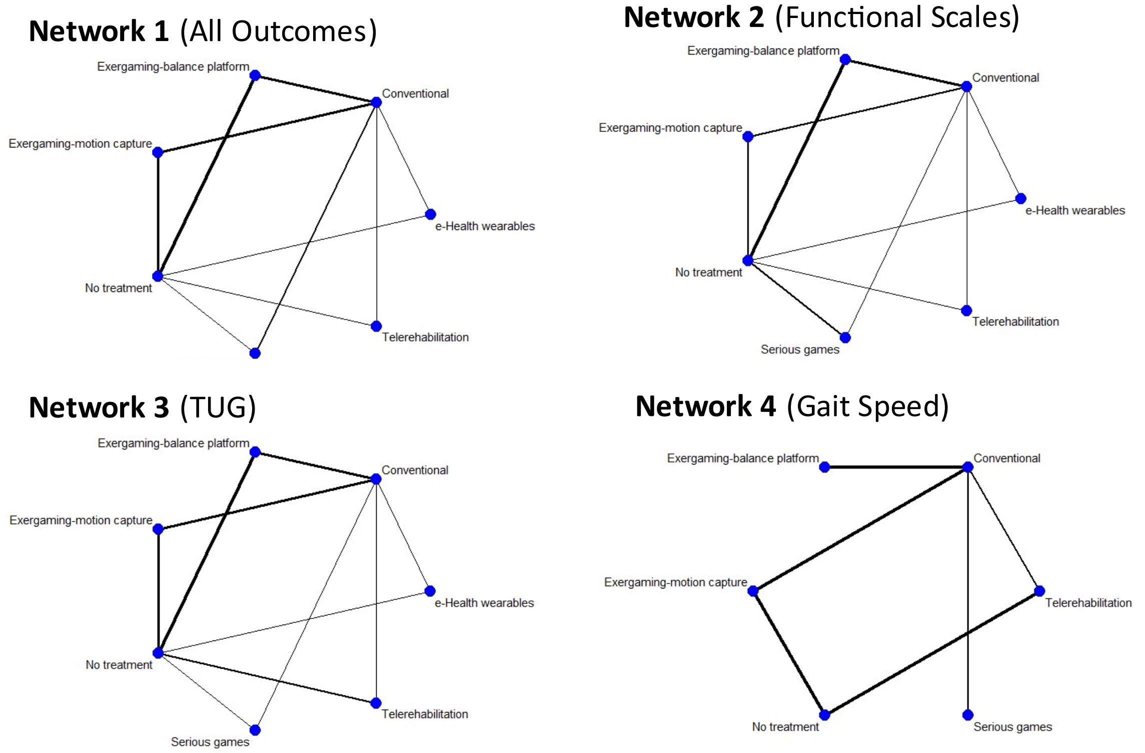

Supplementary Figure 1. Network plot presenting the trial data contributing evidence comparing exercise treatment types for each network.

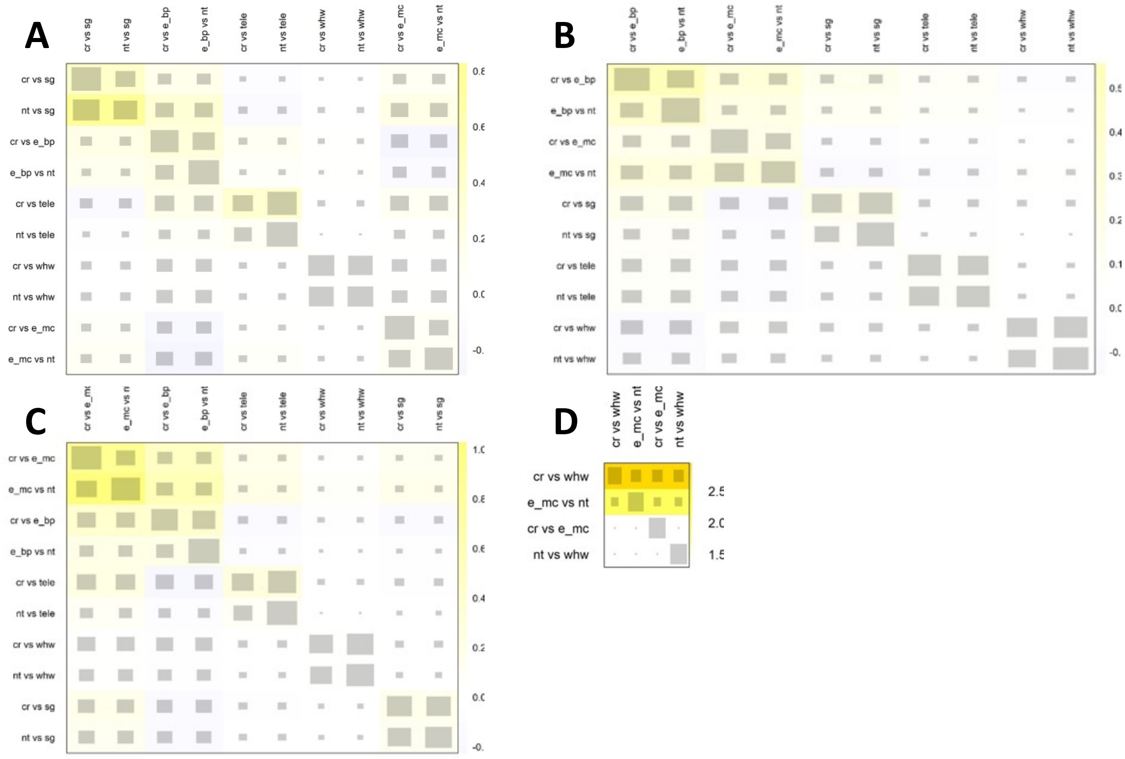

Supplementary Figure 2. Net Heat Plot for Network 1 (All Outcomes)(A), Network 2 (Functional Scales)(B), Network 3 (TUG)(C), and Network 4 (Gait Speed)(D). The gray boxes signify how important a treatment comparison is for the estimation of another treatment comparison. The bigger the box, the more important the comparison. Colored backgrounds indicate the amount of pattern inconsistency in a row that can be attributed to the pattern in a column. The colors of the fields can range from deep red (indicating strong inconsistency) to blue (indicating that the evidence of this design supports the evidence of the row). The yellow colors indicate Qdiff > 0. However, there are no areas of red, so it may be reasonable to conclude that there is no meaningful inconsistency in network.

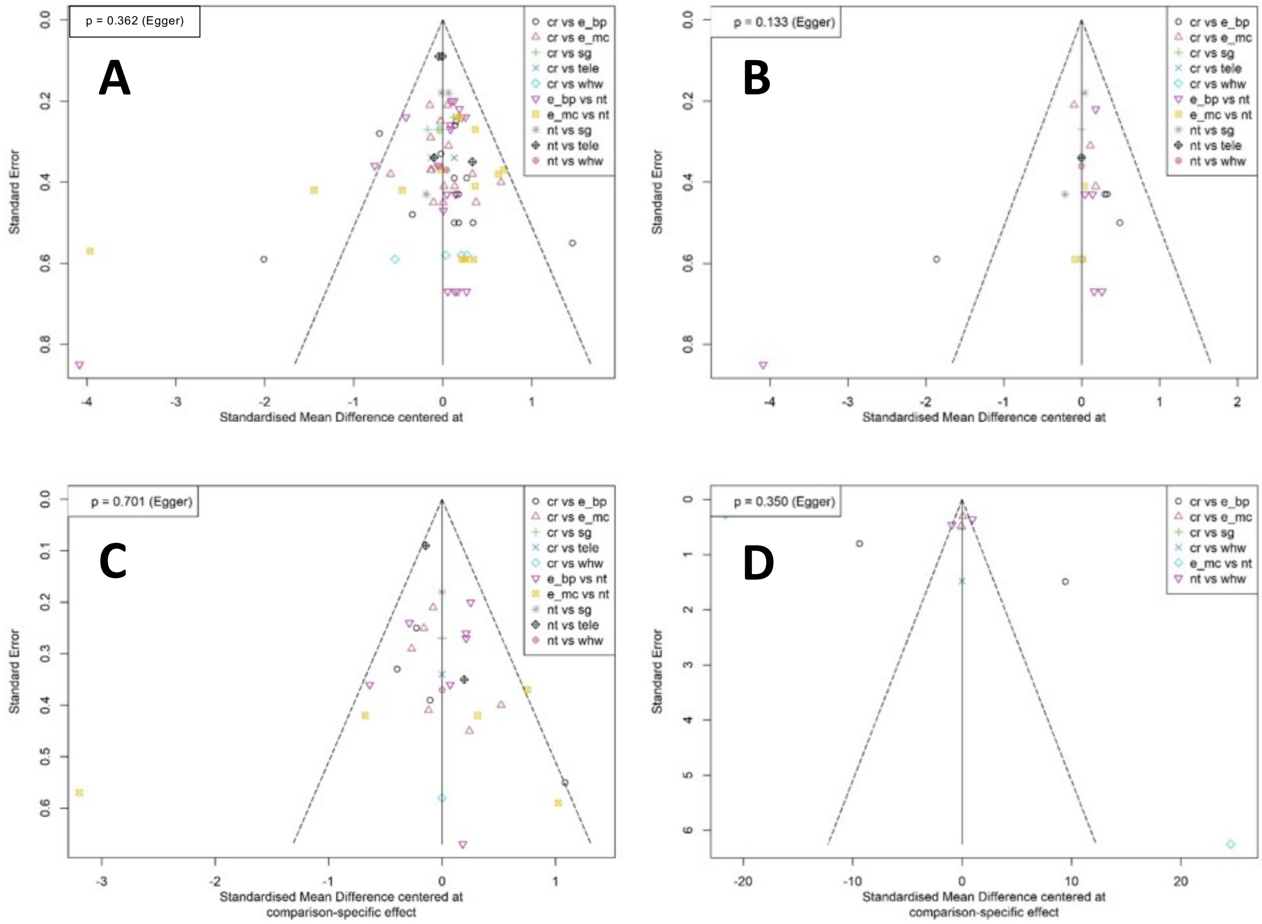

Supplementary Figure 3. Funnel Plots for Network 1 (All Outcomes)(A), Network 2 (Functional Scales)(B), Network 3 (TUG)(C), and Network 4 (Gait Speed)(D).

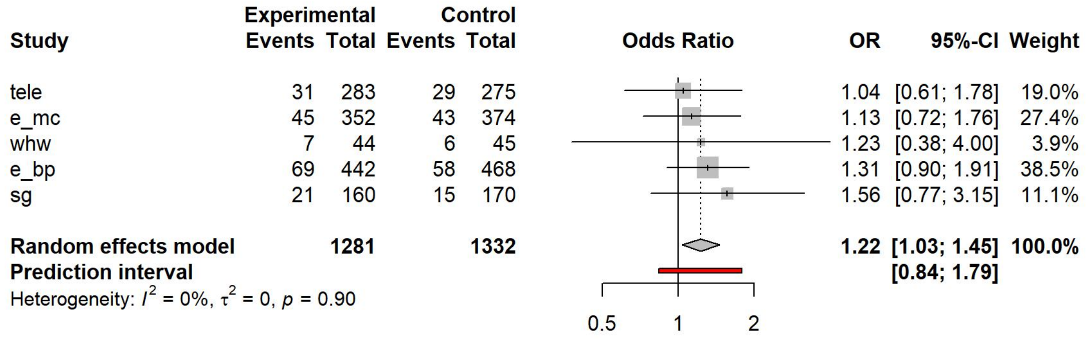

Supplementary Figure 4. Forest plot of the drop-out rates for different treatments compared with conventional exercises. Tele: telerehabilitation; e_mc: exergaming with motion capture; whw: interventions with wearables; e_bp: exergaming with balance platform; sg: other serious gaming.

Supplementary Table 1. Overview of the included studies.

Authors (year)	Sample: population; sample size (n); age, years (mean ± SD)	Interventions	Total number of sessions; duration; session length	Outcomes	Dropouts (n)	Study conclusions
Exergaming with balance platform (n = 22, total participants: 1084)						
Benitez-Lugo et al. (2022)[1]	Community-dwelling older adults, n = 48; 72.6 ± 5.9 years	G1: Exergaming with WBB (n = 28)<br>G2: NT (n = 20)	16 sessions; 8 weeks; 20min	TUG<br>BBS<br>POMA-balance	G1: 2<br>G2: 0	The findings of this study demonstrate that implementing the protocol in older individuals leads to enhancements in memory, precision speed, balance, gait, overall autonomy, and mood.
Hou et al. (2022)[2]	Community-dwelling older adults, n = 84; 67.5 ± 4.6 years	G1: Exergaming with step mat (n = 28)<br>G2: Exergaming with keyboard (n = 29)<br>G3: NT (n = 27)	36 sessions; 12 weeks; 30min	TUG	G1: 5<br>G2: 7<br>G3: 6	Exergame training enhanced lower limb strength and balance in older adults.
Kim and Cho (2022)[3]	Facility-dwelling older adults, n = 36; 79.9 ± 7.1 years	G1: Exergaming with WBB (n = 12)<br>G2: Motor imagery group (n = 12)<br>G3: NT (n = 12)	18 sessions; 6 weeks; 30min	COP	G1: 0<br>G2: 2<br>G3: 0	Study results indicate that the VR program improves balance and reduces fear of falling in older adults
Khanmohammadi et al. (2021)[4]	Community-dwelling older adults, n = 66; 71.5 ± 5.0 years	G1: Exergaming with WBB (n = 33)<br>G2: CE (n = 33)	16 sessions; 5 weeks; 60min	COP	G1: 5<br>G2: 4	Both treatments were effective in improving COP trajectory during the phases of anticipatory and weight transition, while the intervention group was more effective during the phase of locomotor.
Sonthikul et al. (2021)[5]	Community-dwelling older women, n = 34; 63.3 ± 2.3 years	G1: Exergaming on platform (Sensamove®) (n = 17)<br>G2: CE (n = 17)	18 sessions; 6 weeks; 60min	TUG<br>5XSST	G1: 2<br>G2: 2	Active video gaming could not only improve lower limb muscle strength and reaction time, but also increase dynamic balance.
Gallardo-Meza et al. (2020)[6]	Community-dwelling older women, n = 82; 68.7 ± 3.5 years	G1: Exergaming with WBB (n = 41)<br>G2: NT (n = 41)	8 sessions; 4 weeks; 35min	TUG<br>SLS	G1: 6<br>G2: 4	Exergames training improves muscular fitness in older women.
Fakhro et al. (2020)[7]	Community-dwelling older adults, n = 64; 72.2 ± 5.2 years	G1: Exergaming with WBB (n = 34)<br>G2: NT (n = 31)	24 sessions; 8 weeks; 40min	TUG<br>COP	G1: 3<br>G2: 1	Exergaming led to significant improvements in both, dynamic and static balance scores
Morat et al. (2019)[8]	Community-dwelling older adults, n = 51; 69.4 ± 5.6 years	G1: Exergaming with platform (Dividat Senso) (n = 17)<br>G2: Exergaming with unstable platform (Dividat Senso) (n = 17)<br>G3: NT (n = 17)	24 sessions; 8 weeks; 40min	TUG<br>COP	G1: 2<br>G2: 1<br>G3: 0	Exergaming training is an appealing and effective training tool to improve functional balance and calf strength in healthy older adults.
Lim et al. (2017)[9]	Facility-dwelling older adults, n = 20; 79.1 ± 5.9 years	G1: Exergaming with WBB (n = 10)<br>G2: Multicomponent Exergaming with WBB (n = 10)	10 sessions; 5 weeks; 60min	TUG	G1: 0<br>G2: 0	Multicomponent exergaming has superiority for increasing muscle strength and dynamic balance compared to balance exergaming.

Padala et al. (2017)[10]	Community-dwelling older adults, n = 30; 68.0 ± 6.7 years	G1: Exergaming with WBB (n = 15)<br>G2: NT (n = 15)	24 sessions; 8 weeks; 45min	BBS	G1: 2<br>G2: 0	Wii-Fit exercise program is efficacious in improving balance.
Kwok and Pua (2016)[11]	Community-dwelling older adults, n = 80; 69.4 ± 5.6 years	G1: Exergaming with WBB (n = 40)<br>G2: CE (n = 40)	12 sessions; 12 weeks; 20min	TUG	G1: 5<br>G2: 11	Participants who received exergaming intervention had a sustained reduction in fear of falling.
Schättin et al. (2016)[12]	Community-dwelling older adults, n = 29; 79.2 ± 7.3 years	G1: Exergaming with dance platform (n = 15)<br>G2: CE (n = 14)	24 sessions; 8 weeks; 30min	Gait analysis	G1: 2<br>G2: 0	Exergame is able to influence dual-task walking.
Eggenberger et al. (2015)[13]	Community-dwelling older adults, n = 89; 78.8 ± 5.4 years	G1: Exergaming with dance platform (n = 30)<br>G2: Treadmill memory training (n = 29)<br>G3: CE (n = 30)	52 sessions; 26 weeks; 60min	Gait analysis<br>SPPB	G1: 6<br>G2: 7<br>G3: 5	Long term multicomponent cognitive–physical and exclusive physical training programs demonstrated similar potential to counteract age-related decline in physical functioning.
Whyatt et al. (2015)[14]	Facility-dwelling older adults, n = 84; 76.9 ± 6.9 years	G1: Exergaming with WBB (n = 42)<br>G2: NT (n = 42)	10 sessions; 5 weeks; 30min	COP<br>BBS	G1: 2<br>G2: 0	Balance game training had a significant effect on levels of functional balance and balance confidence.
Bieryla and Dold (2013)[15]	Community-dwelling older adults, n = 12; 81.5 ± 4.7 years	G1: Exergaming with WBB (n = 6)<br>G2: NT (n = 6)	9 sessions; 3 weeks; 30min	BBS<br>TUG<br>FAB<br>FRT	G1: 2<br>G2: 1	Exergaming improved BBS scores 1 month after the intervention concluded.
Jorgensen et al. (2013)[16]	Community-dwelling older adults, n = 58; 76.4 ± 6.6 years	G1: Exergaming with WBB (n = 28)<br>G2: NT (n = 30)	20 sessions; 10 weeks; 35min	TUG	G1: 3<br>G2: 0	Exergaming improvements in maximal leg muscle strength. Static bilateral postural balance remained unaltered with Wii training.
Singh et al. (2013)[17]	Community-dwelling older women, n = 38; 77.2 ± 7.0 years	G1: Exergaming with WBB (n = 19)<br>G2: CE (n = 19)	12 sessions; 6 weeks; 40min	OPI<br>TUG	G1: 1<br>G2: 1	Balance-focused VR games improved the agility, balance and functional mobility.
Franco et al. (2012)[18]	Facility-dwelling older adults, n = 32; 78.3 ± 6.0 years	G1: Exergaming with WBB (n = 11)<br>G2: CE (n = 11)<br>G3: NT (n = 10)	6 sessions; 3 weeks; 15min	BBS<br>POMA	NI	The interventions failed to significantly increase balance.
Pichierri et al. (2012)[19]	Facility-dwelling older adults, n = 31; 86.2 ± 4.6 years	G1: CE + Exergaming with dance pad (n = 15)<br>G2: CR (n = 16)	24 sessions; 12 weeks; 40min	Gait analysis<br>FPA<br>PPA<br>FES-I	G1: 5<br>G2: 5	Enhancements in walking performance under dual task conditions were observed for the dance video game group only.
Pluchino et al. (2012)[20]	Community-dwelling older adults, n = 40; 72.5 ± 8.4 years	G1: Exergaming with WBB (n = 12)<br>G2: Tai Chi (n = 14)<br>G3: CE (n = 14)	16 sessions; 8 weeks; 60min	SLS<br>TUG<br>FRT<br>POMA<br>COP	G1: 4<br>G2: 3<br>G3: 6	Wii balance program is as effective as formalized training programs at improving balance in healthy community-dwelling older persons.

Rendon et al. (2012)[21]	Community-dwelling older adults, n = 40; 84.5 ± 5.2 years	G1: Exergaming with WBB (n = 20)<br>G2: NT (n = 20)	18 sessions; 6 weeks; 45min	TUG	G1: 4<br>G2: 2	Virtual reality gaming provides clinicians with a useful tool for improving dynamic balance and balance confidence in older adults.
Toulotte et al. (2012)[22]	Community-dwelling older adults, n = 36; 75.1 ± 10.2 years	G1: Exergaming with WBB (n = 9)<br>G2: Exergaming with WBB+CE (n = 9)<br>G3: CE (n = 9)<br>G4: NT (n = 9)	20 sessions; 6 weeks; 45min	SLS<br>POMA	NI	All intervenes improved participant's balance. In addition, CR and combined CR and exergaming increased their dynamic balance.
Exergaming with motion capture (n = 17, total participants: 912)						
AL-Emrany et al. (2021)[23]	Community-dwelling older adults, n = 35; 67.1 ± 1.8 years	G1: Exergaming with Kinect (n = 25)<br>G2: CE (n = 10)	30 sessions; 6 weeks; 30min	OSI<br>FRT	G1: 0<br>G2: 0	Virtual reality training improved the overall postural stability.
Babadi and Daneshmandi (2021)[24]	Facility-dwelling older adults, n = 36; 66.9 ± 4.5 years	G1: Exergaming with Kinect (n = 12)<br>G2: CE (n = 12)<br>G3: NT (n = 12)	27 sessions; 9 weeks; 60min	TUG<br>FAB<br>SLS	G1: 0<br>G2: 0<br>G3: 0	In both groups (exergaming, CR), SLS with open and closed eyes, FRT, TUG, and FABS were significantly improved. After the intervention, changes in both groups were similar, which indicates that neither exergaming and CR training methods were superior to the other.
De Lima et al. (2021)[25]	Community-dwelling older adults, n = 38; 69.4 ± 5.6 years	G1: Exergaming with Kinect (n = 19)<br>G2: NT (n = 19)	18 sessions; 6 weeks; 60min	TUG	G1: 5<br>G2: 4	Exergaming did not have significant influence on TUG.
Moreira et al. (2021)[26]	Community-dwelling older adults, n = 99; 70.8 ± 5.1 years	G1: Exergaming with Kinect (n = 49)<br>G2: CE (n = 50)	36 sessions; 12 weeks; 50min	TUG<br>Gait analysis	G1: 17<br>G2: 16	Exergame or multicomponent exercise training routines were effective for improving balance.
Sadeghi et al. (2021)[27]	Community-dwelling older men, n = 64; 71.8 ± 5.9 years	G1: Exergaming with Kinect (n = 16)<br>G2: CE + Exergaming (n = 16)<br>G3: CE (n = 16)<br>G4: NT (n = 16)	24 sessions; 8 weeks; 40min	TUG<br>SLS<br>Tandem Stance	G1: 1<br>G2: 2<br>G3: 2<br>G4: 1	Group with mixed CR and Exergaming elicited the greatest improvements in balance. Exergaming alone group exhibited better balance and functional mobility relative to CR and NT.
Chen et al. (2020)[28]	Community-dwelling older adults, n = 28; 73.6 ± 4.1 years	G1: Tai-Chi exercise with Kinect (n = 14)<br>G2: CE (n = 14	24 sessions; 8 weeks; 30min	BBS<br>TUG<br>FRT	NI	VR-augmented training system could achieve training goals more readily than use of traditional full course.
Yang et al. (2020)[29]	Community-dwelling older adults, n = 20; 68.7 years	G1: Exergaming with Kinect (n = 10)<br>G2: CE (n = 10)	10 sessions; 5 weeks; 45min	TUG<br>RT<br>SLS	G1: 0<br>G2: 0	Kinect exercise was more effective in terms of overall balance ability, and was particularly beneficial to FRT enhancement in comparison with traditional exercise.
Phirom et al. (2020)[30]	Community-dwelling older adults, n = 40; 69.8 ± 3.8 years	G1: Exergaming with Kinect + LED projector (n = 20)<br>G2: NT (n = 20)	36 sessions; 12 weeks; 60min	PPA<br>TUG	G1: 1<br>G2: 0	Interactive physical-cognitive, game-based training program could improve speed processing and body sway.
Bacha et al. (2018)[31]	Community-dwelling older adults, n = 46; 69.3 ± 5.3 years	G1: Exergaming with Kinect (n = 23)<br>G2: CE (n = 23)	14 sessions; 7 weeks; 60min	Mini-BESTest<br>FGA	G1: 2<br>G2: 2	Both interventions provided positive effects on postural control and gait.

Htut et al. (2018)[32]	Facility-dwelling older adults, n = 84; 72.2 ± 5.2 years	G1: Exergaming with Kinect (n = 21)<br>G2: CE (n = 21)<br>G3: Cognitive training (n = 21)<br>G4: NT (n = 21)	24 sessions; 8 weeks; 30min	TUG<br>BBS<br>5XSST<br>FES-I	G1: 0<br>G2: 0<br>G3: 0<br>G4: 0	Exergaming enhanced physical performance. Older persons favored exergaming and cognitive training as these routines were considered more enjoyable.
Ordnung et al. (2017)[33]	Community-dwelling older adults, n = 30; 69.2 ± 5.5 years	G1: Exergaming with Kinect (n = 15)<br>G2: NT (n = 15)	12 sessions; 6 weeks; 60min	COP	G1: 1<br>G2: 0	Within-group comparison revealed improvements in sensorimotor and cognitive tasks in exergaming group, while NT only showed an improvement in a static balance test.
Bieryla (2015)[34]	Community-dwelling older adults, n = 13; 82.3 ± 4.6 years	G1: Exergaming with Kinect (n = 6)<br>G2: NT (n = 7)	9 sessions; 3 weeks; 30min	BBS<br>TUG<br>FAB<br>FRT	G1: 1<br>G2: 0	A training program using the Kinect with commercially available games was feasible with old adults.
Chow and Mann (2015)[35]	Community-dwelling older adults, n = 20; 69.1 ± 4.1 years	G1: Exergaming with Kinect (n = 10)<br>G2: NT (n = 10)	14 sessions; 2 weeks; 30min	FRT<br>SLS<br>TUG	NI	Cyber-golfing may be considered a therapeutic activity for static-balance training in geriatric care.
Gschwind et al. (2015)[36]	Community-dwelling older adults, n = 148; 80.9 ± 6.6 years	G1: Exergaming with Kinect (n = 29)<br>G2: Exergaming with balance board (n = 47)<br>G3: NT (n = 72)	48 sessions; 16 weeks; 20min	PPA<br>5XSST<br>TUG	G1: 5<br>G2: 8<br>G3: 10	The findings suggest that it is feasible for older people to conduct an unsupervised exercise program at home using exergames.
Karahan et al. (2015)[37]	Community-dwelling older adults, n = 100; 71.4 ± 5.4 years	G1: Exergaming with Kinect (n = 54)<br>G2: CE (n = 46)	30 sessions; 6 weeks; 30min	BBS<br>TUG	G1: 6<br>G2: 4	Exergaming had positive effects on balance and functional walking.
Lee et al. (2015)[38]	Community-dwelling older adults, n = 54; 68.2 ± 4.4 years	G1: Exergaming with Kinect (n = 26)<br>G2: CE (n = 28)	24 sessions; 8 weeks; 60min	TUG	G1: 4<br>G2: 3	Exergaming did not improve TUG score.
Sato et al. (2015)[39]	Community-dwelling older adults, n = 57; 69.2 ± 5.4 years	G1: Exergaming with Kinect (n = 29)<br>G2: NT (n = 28)	24 sessions, NI; 40min	Gait analysis<br>BBS<br>FRT	G1: 1<br>G2: 2	The Kinect-based exergame developed in this study was found to be effective in improving walking, muscular strength, and balance in older adults.
Other serious games (n = 7, total participants: 452)						
Liepa et al (2022)[40]	Community-dwelling older adults, n = 44; 81.5 ± 4.7 years	G1: Serious games with HMD (n = 15)<br>G2: Gaming on laptop (n = 15)<br>G3: NT (n = 15)	18 sessions; 9 weeks; 25min	SPPB	G1: 0<br>G2: 0<br>G3: 0	Exergaming did not affect the SPPB results.
Gonçalves et al. (2021)[41]	Community-dwelling older adults, n = 37; 68.3 ± 4.7 years	G1: Serious games with motion capture (n = 19)<br>G2: CE (n = 18)	24 sessions; 12 weeks; 60min	FAB<br>TUG	G1: 4<br>G2: 2	Integrating personalized exergames designed for multidimensional fitness training in traditional settings can be an effective strategy to enhance older adults' motor performance.
Lee (2021)[42]	Community-dwelling older adults, n = 56; 80.2 ± 6.5 years	G1: Serious games with VR gait training (n = 28)<br>G2: CE (n = 28)	20 sessions; 4 weeks; 50min	SLS<br>BBS<br>FRT<br>TUG	G1: 0<br>G2: 0	Serious games with VR gait training have shown to improve balance and gait ability in older adults.

				Gait analysis		
Li et al. (2020)[43]	Community-dwelling older adults, n = 20; 73.1 ± 7.4 years	G1: Serious games with HMD (n = 10)<br>G2: NT (n = 10)	12 sessions; 4 weeks; 45min	SLS	G1: 0<br>G2: 0	The results show that video game training significantly increased one-leg balance time for the open-eyes condition.
Park and Yim (2016)[44]	Facility-dwelling older adults, n = 72; 73.5 ± 2.9 years	G1: CR + 3D VR Kayak Program (n = 36)<br>G2: CE (n = 36)	12 sessions; 6 weeks; 50min	COP	NI	Virtual kayaking reduced risk of cognitive impairment, upper extremity strength, and balance ability.
Gschwind et al. (2015)[45]	Community-dwelling older adults, n = 153; 74.7 ± 6.3 years	G1: CE + serious games with motion capture (n = 78)<br>G2: NT (n = 75)	48 sessions; 16 weeks; 40min	PPA<br>TUG<br>SPPB	G1: 15<br>G2: 13	The exergame exercise program reduced physiological fall risk in the study sample.
Duque et al. (2013)[46]	Community-dwelling older adults, n = 70; 77.2 ± 7.0 years	G1: Serious games with HMD (n = 30)<br>G2: NT (n = 40)	12 sessions; 6 weeks; 20min	Gait analysis<br>COP	G1: 2<br>G2: 0	VR training is an effective and intervention to improve balance, increase confidence, and prevent falls in older adults.
Interventions with wearables (n = 4, total participants: 98)						
Bao et al. (2022)[47]	Community-dwelling older adults, n = 16; 75.4 ± 4.7 years	G1: Home training with wearable smartphone balance trainer (n = 8)<br>G2: CE (n = 8)	24 sessions; 8 weeks; NI	CDP<br>Mini-BESTest<br>5XSST<br>TUG<br>FRT	G1: 2<br>G2: 2	Both groups demonstrated significant improvements in their SOT composite and Mini-BESTest scores. However, only the group that trained with vibrotactile SA maintained a minimal detectable change six months following completion of the balance training protocol.
Adcock et al. (2020)[48]	Community-dwelling older adults, n = 37; 73.9 ± 6.4 years	G1: Multicomponent gaming with wearables (Active@Home) (n = 18)<br>G2: NT (n = 19)	48 sessions; 16 weeks; 40min	Gait analysis<br>SPPB	G1: 3<br>G2: 3	No significant improvements got evident for any of the measured physical functions (gait parameters, muscle strength, balance).
Bao et al. (2018)[49]	Community-dwelling older adults, n = 12; 75.8 ± 5.2 years	G1: Home training with wearable smartphone balance trainer (n = 6)<br>G2: CE (n = 6)	24 sessions; 8 weeks; 45min	Mini-BESTest<br>5XSST<br>FRT<br>TUG<br>Gait analysis	G1: 0<br>G2: 0	In-home balance training with improved balance performance of older adults.
Schwenk et al. (2014)[50]	Facility-dwelling older adults, n = 33; 84.6 ± 6.9 years	G1: Serious games with wearables (n = 17)<br>G2: NT (n = 16)	8 sessions; 4 weeks; 45min	COM<br>AST<br>TUG<br>Gait analysis	G1: 2<br>G2: 1	Results of this proof-of-concept study suggest that older adults at risk of falling can benefit from the balance training program.
Telerehabilitation (n = 3, total participants: 619)						
Delbaere et al (2021)[51]	Community-dwelling older adults, n = 503; 69.8 ± 3.8 years	G1: StandingTall programme (n = 254)<br>G2: NT (n = 249)	104 weeks; NI; 20min	TUG<br>5XSST<br>SPPB	G1: 29<br>G2: 26	The StandingTall balance exercise programme did not significantly affect selected outcomes.
Yerlikaya et al. (2021)[52]	Community-dwelling older adults, n = 52; 72.5 ± 6.9 years	G1: Rehabilitation delivered remotely (n = 18)<br>G2: CE (n = 17)<br>G3: NT (n = 17)	24 sessions; 8 weeks, 40min	TUG<br>BBS<br>Acceleromete r	G1: 0<br>G2: 1<br>G3: 1	Although non-supervised home exercise and tele-exercise were effective on the improvement of balance, mobility and reduction of fall risk in older individuals, this effect was found to be greater in the tele group.

Wu et al. (2010)[53]	Community-dwelling older adults, n = 64; 69.8 ± 3.8 years	G1: Tai Chi exercise program delivered remotely (n = 22)<br>G2: Tai Chi exercise program in community center (n = 20)<br>G3: Tai Chi exercise program conducted at home (n = 22)	45 sessions; 15 weeks; 60min	TUG<br>SLS<br>COP	G1: 2<br>G2: 1<br>G3: 10	Compared with home, Tele and Comm groups are better in exercise compliance, fall reduction and balance and health improvements.
Other interventions (n = 2, total participants: 108)						
Lee (2023)[54]	Community-dwelling older adults, n = 60; 79.7 ± 3.2 years	G1: Exergaming with Switch (n = 30)<br>G2: NT (n = 30)	24 sessions; 8 weeks; 50min	TUG<br>SLS<br>BBS<br>FRT<br>SPPB	G1: 2<br>G2: 1	Eight-week home-based Switch exergame program can lead to noteworthy enhancements in physical function, fall efficacy, depression, and health-related quality of life among community-dwelling older adults.
Zhao et al. (2022)[55]	Community-dwelling older adults, n = 48; 65.7 ± 3.8 years	G1: Exergaming with Switch (n = 24)<br>G2: NT (n = 24)	36 sessions; 12 weeks; 55min	COP	G1: 2<br>G2: 8	Video game exercise as a feasible, safe, and effective training method for improving community older adults' healthy, promoting group cohesion, and increasing motivation to exercise.

G: Group; VR: Virtual Reality; HMD: Head-Mounted Display; WBB: Wii Balance Board; CE: Conventional Exercises; NT: No Treatment; NI: No Information; FPA: Foot Placement Accuracy; PPA: Physiological Profile Assessment; FES-I: Falls Efficacy Scale International; COP: Center of Pressure; BBS: Berg Balance Scale; TUG: Timed Up And Go Test; FRT: Functional Reach Test; 5XSST: Five Time Sit To Stand Test; DGI: Dynamic Gait Index; DVA: Dynamic Visual Acuity; SLS: Single Leg Stance; OSI: Overall Stability Index; SPPB: Short Physical Performance Battery'; OPI: Overall Performance Index; Mini-BESTest: Mini-Balance Evaluation Systems Test; FGA: Functional Gait Assessment; FAB: Fullerton Advanced Balance Scale; SPPB: Short Physical Performance Battery; COM: Center of Mass; AST: Alternate Step Test; POMA: Tinetti Performance Oriented Mobility Assessment; CDP: Computerized Dynamic Posturography.

Supplementary Table 2. Effect Estimates (indirect and direct).

Effect Estimate for Network 1 (All Outcomes)						
CE	-0.15 [-0.36; 0.07]	-0.02 [-0.21; 0.16]	.	0.06 [-0.18; 0.30]	0.13 [-0.38; 0.64]	-0.04 [-0.63; 0.54]
-0.06 [-0.23; 0.10], P = 0.47	EBP	.	-0.14 [-0.30; 0.02]	.	.	.
0.01 [-0.15; 0.16], P = 0.94	0.07 [-0.12; 0.26]; P = 0.46	EMC	-0.20 [-0.41; 0.01]	.	.	.
0.16 [-0.33; 0.01], P = 0.06	-0.09 [-0.23; 0.05], P = 0.22	-0.16 [-0.33; 0.01]; P = 0.06	NT	-0.05 [-0.35; 0.25]	0.03 [-0.18; 0.25]	0.03 [-0.51; 0.57]
-0.04 [-0.24; 0.15], P = 0.67	0.02 [-0.21; 0.25]; P = 0.86	-0.05 [-0.28; 0.18]; P = 0.68	0.11 [-0.10; 0.32], P = 0.31	SG	.	.
-0.08 [-0.32; 0.16], P = 0.50	-0.02 [-0.26; 0.22]; P = 0.88	-0.09 [-0.34; 0.16]; P = 0.49	0.07 [-0.13; 0.27], P = 0.48	-0.04 [-0.32; 0.24]; P = 0.78	TELE	.
-0.09 [-0.49; 0.32], P = 0.68	-0.02 [-0.44; 0.40]; P = 0.91	-0.09 [-0.52; 0.33]; P = 0.67	0.07 [-0.34; 0.47], P = 0.75	-0.04 [-0.48; 0.40]; P = 0.84	-0.00 [-0.45; 0.44]; P = 0.98	EHI
Effect Estimate for Network 2 (Functional Scales)						
CE	-0.30 [-0.76; 0.17]	-0.07 [-0.39; 0.24]	.	0.00 [-0.53; 0.53]	0.00 [-0.67; 0.67]	-0.58 [-1.74; 0.58]
-0.10 [-0.45; 0.24]; P = 0.55	EBP	.	-0.13 [-0.45; 0.19]	.	.	.
-0.12 [-0.40; 0.17]; P = 0.42	-0.01 [-0.42; 0.39]; P = 0.95	EMC	0.13 [-0.45; 0.70]	.	.	.
-0.14 [-0.46; 0.18]; P = 0.38	-0.04 [-0.32; 0.25]; P = 0.79	-0.03 [-0.40; 0.34]; P = 0.89	NT	-0.03 [-0.35; 0.30]	-0.06 [-0.73; 0.61]	-0.01 [-0.72; 0.70]
-0.12 [-0.49; 0.24]; P = 0.50	-0.02 [-0.40; 0.36]; P = 0.92	-0.01 [-0.43; 0.42]; P = 0.98	0.02 [-0.27; 0.31]; P = 0.90	SG	.	.
-0.10 [-0.60; 0.40]; P = 0.69	0.00 [-0.54; 0.55]; P = 0.99	0.02 [-0.54; 0.57]; P = 0.96	0.04 [-0.46; 0.54]; P = 0.87	0.02 [-0.53; 0.57]; P = 0.94	TELE	.
-0.27 [-0.91; 0.38]; P = 0.41	-0.16 [-0.82; 0.49]; P = 0.62	-0.15 [-0.83; 0.53]; P = 0.66	-0.13 [-0.73; 0.48]; P = 0.68	-0.15 [-0.81; 0.52]; P = 0.67	-0.17 [-0.94; 0.60] ; P = 0.67	EHI
Effect Estimate for Network 3 (TUG)						
CE	0.23 [-0.44; 0.89]	0.11 [-0.42; 0.63]	.	0.02 [-1.21; 1.25]	0.26 [-1.03; 1.55]	-0.01 [-1.60; 1.58]
0.02 [-0.48; 0.53]; P = 0.93	EBP	.	-0.26 [-0.75; 0.22]	.	.	.
0.35 [-0.10; 0.79]; P = 0.13	0.33 [-0.23; 0.88]; P = 0.25	EMC	-0.97 [-1.54; -0.41]	.	.	.
-0.35 [-0.84; 0.14]; P = 0.16	-0.37 [-0.80; 0.06]; P = 0.09	-0.70 [-1.16; -0.23]; P = 0.003	NT	-0.07 [-1.23; 1.09]	0.17 [-0.67; 1.02]	0.07 [-1.25; 1.39]
-0.21 [-1.09; 0.67]; P = 0.64	-0.23 [-1.16; 0.70]; P = 0.62	-0.56 [-1.49; 0.37]; P = 0.24	0.14 [-0.74; 1.01]; P = 0.76	sg	.	.
-0.04 [-0.83; 0.74]; P = 0.91	-0.06 [-0.88; 0.75]; P = 0.87	-0.39 [-1.21; 0.42]; P = 0.35	0.31 [-0.42; 1.03]; P = 0.41	0.17 [-0.94; 1.27]; P = 0.76	TELE	.
-0.17 [-1.22; 0.89]; P = 0.75	-0.19 [-1.28; 0.90]; P = 0.73	-0.52 [-1.60; 0.57]; P = 0.35	0.18 [-0.85; 1.22]; P = 0.73	0.04 [-1.28; 1.36]; P = 0.95	-0.13 [-1.37; 1.11]; P = 0.84	EHI
Effect Estimate for Network 4 (Gait Speed)						
CE	5.01 [-18.64; 28.66]	2.12 [-21.48; 25.71]	-	-4.97 [-38.35; 28.41]	-	-6.66 [-40.15; 26.83]
5.01 [-18.64; 28.66]; P = 0.68	EBP	-	-	-	-	-
-3.74[-24.89; 17.40]; P = 0.73	-8.75 [-40.48; 22.97]; P = 0.59	EMC	22.14 [-2.18; 46.47]	-	-	-
12.18 [-13.94; 38.29]; P = 0.36	7.17 [-28.06; 42.40]; P = 0.69	15.92 [-5.71; 37.55]; P = 0.15	NT	-	-	-1.18 [-24.78; 22.41]
-4.97 [-38.35; 28.41]; P = 0.77	-9.98 [-50.89; 30.93]; P = 0.63	-1.23 [-40.74; 38.29]; P = 0.95	-17.15 [-59.53; 25.23]; P = 0.43	SG	-	-
5.14 [-20.88; 31.15]; P = 0.70	0.13 [-35.03; 35.28]; P = 0.99	8.88 [-17.24; 34.99]; P = 0.50	-7.04 [-28.19; 14.10]; P = 0.51	10.11 [-32.21; 52.42]; P = 0.64	-	EHI

Results of the effect of intervention in each row compared with intervention in each column. The upper triangle of the table displays the pooled effect sizes of the available direct comparisons. Because we do not have direct evidence for all comparisons, some fields in the upper triangle remain empty (.). The lower triangle of the matrix contains the estimated effect sizes for each comparison, even those for which only indirect evidence was available. Mean difference <0 favors the treatment in the column, mean difference >0 favors the treatment in the row.

Supplementary notes

List of studies excluded at full-text screening stage, with brief reasons

Incorrect study type:

1. Balance, attention and concentration improvements following an exergame training program in elderly. **Reason: conference abstract.**
2. Effects Of a New Computerized Motor-Cognitive Memory Training in Older Persons. **Reason: supplementary abstract.**
3. Benefits of a virtual environment program at the level of functional physical fitness in non-institutionalized elderly. **Reason: supplementary abstract.**
4. Effects of Remotely Supervised Physical Activity on Health Profile in Frail Older Adults: A Randomized Controlled Trial Protocol. **Reason: study protocol.**
5. Exergaming as a tool to enhance strength, balance, gait, mobility, participation, self-efficacy for exercise and adherence in older adults. **Reason: supplementary abstract.**
6. Fast-paced videogame training improves balance under dynamic visual conditions in older adults. **Reason: conference abstract.**
7. The effect of wii-based interventions on physical, cognitive and social functioning among pre-frail elderly persons. **Reason: conference poster.**
8. Virtual Reality Gaming as a Tool for Rehabilitation in Physical Therapy. **Reason: incorrect publication type.**
9. Progressive functional wii-hab in pre-frail older adults. **Reason: conference paper.**
10. Protocol of a 12-month multifactorial eHealth programme targeting balance, dual-tasking and mood to prevent falls in older people: the StandingTall + randomised controlled trial. **Reason: study protocol.**
11. A Pilot Study of an In-Home Multicomponent Exergame Training for Older Adults: Feasibility, Usability and Pre-Post Evaluation. **Reason: not RCT.**
12. Balance training monitoring and individual response during unstable vs. stable balance Exergaming in elderly adults: findings from a randomized controlled trial. **Reason: not a RCT.**
13. Is virtual reality exercise effective in reducing falls among older adults with a history of falls? **Reason: supplementary abstract.**
14. Older Adults' Experience of an Exergaming Intervention to Improve Balance and Prevent Falls: A Nested Explanatory Qualitative Study. **Reason: not RCT.**
15. Feasibility of Virtually Delivering Functional Fitness Assessments and a Fitness Training Program in Community-Dwelling Older Adults. **Incorrect study design**

Incorrect outcomes:

1. Web-based Intervention to Promote Physical Activity by Sedentary Older Adults: Randomized Controlled Trial. **Reason: incorrect outcomes.**
2. The effect of exergaming on knee proprioception in older men: A randomized controlled trial. **Reason: incorrect outcome.**
3. Video game and motor-cognitive dual-task training could be suitable treatments to improve dual-task interference in older adults. **Reason: incorrect outcome.**

Incorrect intervention:

1. A Novel Video Game–Based Device for Measuring Stepping Performance and Fall Risk in Older People. **Reason: no intervention.**
2. Effects of augmented reality-based Otago exercise on balance, gait, and physical factors in elderly women to prevent falls: a randomized controlled trial. **Reason: cannot identify the augmented reality source.**
3. Acute effects of virtual reality treadmill training on gait and cognition in older adults: A randomized controlled trial. **Reason: RCT with one intervention session.**
4. Wii-hab for pre-frail older adults. **Reason: incorrect intervention (additional weight vest in exergaming group).**

Incorrect participants:

1. Comparison of exergaming and vestibular training on gaze stability, balance, and gait performance of older adults: a single blind randomized control trial. **Reason: incorrect participants.**
2. Effects of Activity Tracker Use With Health Professional Support or Telephone Counseling on Maintenance of Physical Activity and Health Outcomes in Older Adults: Randomized Controlled Trial. **Reason: group initially trained.**

3. Effects of an Interactive Computer Game Exercise Regimen on Balance Impairment in Frail Community-Dwelling Older Adults: A Randomized Controlled Trial. **Reason: incorrect participants.**
4. Effects of Kinect-based exergaming on frailty status and physical performance in prefrail and frail elderly: a randomized controlled trial. **Reason: Incorrect participants.**
5. Effects of Wii Fit exer-gaming on balance and gait in elderly population: A randomized control trial. **Reason: incorrect participants.**
6. Fall-Prone Older People's Attitudes towards the Use of Virtual Reality Technology for Fall Prevention. **Reason: incorrect participants.**
7. Feasibility, safety, acceptability, and functional outcomes of playing Nintendo Wii Fit PlusTM for frail older adults: A randomized feasibility clinical trial. **Reason: incorrect participants.**
8. Virtual reality exercise to improve balance control in older adults at risk of falling. **Reason: incorrect participants.**
9. Virtual reality system based on Kinect for the elderly in fall prevention. **Reason: incorrect participants.**
10. Increases in muscle strength and balance using a resistance training program administered via a telecommunications system in older adults. **Reason: incorrect participants.**
11. The effect of virtual reality-based balance training on motor learning and postural control in healthy adults: a randomized preliminary study. **Reason: incorrect participants.**
12. Effect of virtual running with exercise on functionality in pre-frail and frail elderly people: randomized clinical trial. **Reason: incorrect participants.**
13. Is Balance Training Using Biofeedback Effective in the Prophylaxis of Falls in Women over the Age of 65? **Reason: incorrect participants.**
14. A Comparative Analysis of Wii Fit Training (Wft) Versus Reactive Balance Training (Rbt) For Among Elderly Population. **Reason: incorrect participants.**

Other:

1. Effects of a physical activity program using exergame with elderly women. **Reason: article in Korean.**

Full search strategy

PubMed

((((((((((((((((((((((((((((((((((((("Aged"[MeSH Terms] OR "older"[All Fields] OR "elder*"[All Fields] OR "senior"[All Fields]) NOT "stroke"[All Fields]) NOT "parkinson's"[All Fields]) NOT "parkinson"[All Fields]) NOT "Chronic Obstructive Pulmonary Disease"[All Fields]) NOT "COPD"[All Fields]) NOT "cardiac"[All Fields]) NOT "pulmonary"[All Fields]) NOT "neurologic"[All Fields]) NOT "hemiplegia"[All Fields]) NOT "paraplegia"[All Fields]) NOT "neuropathy"[All Fields]) NOT "arthroplasty"[All Fields]) NOT "kidney"[All Fields]) NOT "vestibular loss"[All Fields]) NOT "amblyopia"[All Fields]) NOT "fracture"[All Fields]) NOT "Meniere's"[All Fields]) NOT "breast cancer"[All Fields]) NOT "cancer"[All Fields]) NOT "replacement"[All Fields]) NOT "Alzheimer's"[All Fields]) NOT "dementia"[All Fields]) NOT "down syndrome"[All Fields]) NOT "low back pain"[All Fields]) NOT "amputation"[All Fields]) NOT "Huntington disease"[All Fields]) NOT "multiple sclerosis"[All Fields]) NOT "insomnia"[All Fields]) NOT "Fibromyalgia"[All Fields]) NOT "cochleovestibular loss"[All Fields]) NOT "osteoarthritis"[All Fields]) NOT "osteoporosis"[All Fields]) NOT "surgery"[All Fields]) NOT "Diabetes"[All Fields]) NOT "review"[All Fields]) NOT "meta-analysis"[All Fields]) NOT "study protocol"[All Fields]) NOT "quasi-randomized"[All Fields]) NOT "Cerebral palsy"[All Fields]) AND ("Virtual Reality"[MeSH Terms] OR "Virtual Reality Exposure Therapy"[MeSH Terms] OR "Exergaming"[MeSH Terms] OR "game-based rehabilitation"[All Fields] OR "head mounted display"[All Fields] OR "commercial video gam*"[All Fields] OR "video gam*"[All Fields] OR "Virtual Reality"[All Fields] OR "Nintendo"[All Fields] OR "WiiPD"[All Fields] OR "WII fit"[All Fields] OR "Wii-hab"[All Fields] OR "Xbox"[All Fields] OR "PlayStation"[All Fields] OR "Kinect"[All Fields] OR "VR"[All Fields] OR "exergam*"[All Fields] OR "virtual reality exercis*"[All Fields] OR "active video gam*"[All Fields] OR "serious gam*"[All Fields] OR "augmented reality"[All Fields] OR "mixed reality"[All Fields] OR "Telemedicine"[MeSH Terms] OR "Telemedicine"[All Fields] OR "Telehealth"[All Fields] OR "Telerehabilitation"[All Fields] OR "videoconferenc*"[All Fields] OR "Teletherapy"[All Fields] OR "teleconferenc*"[All Fields] OR "Telepractice"[All Fields] OR "teletreatment*"[All Fields] OR "Virtual conference"[All Fields] OR "ehealth"[All Fields]) AND ("gait"[MeSH Terms] OR "Postural Balance"[MeSH Terms] OR "gait analysis"[MeSH Terms] OR "Walk Test"[MeSH Terms] OR "postural stability"[All Fields] OR "balance"[All Fields] OR "gait"[All Fields] OR "posture"[All Fields] OR "force plate"[All Fields] OR "tug"[All Fields] OR "up and go"[All Fields] OR "bestest"[All Fields] OR "bbs"[All Fields] OR "berg balance scale"[All Fields] OR "kistler"[All Fields] OR "Dynamic Gait Index"[All Fields] OR "stability"[All Fields] OR "leg stance"[All Fields] OR "SLS"[All Fields] OR "Functional reach test"[All Fields] OR "FRT"[All Fields] OR "Fullerton Advance Balance Scale"[All Fields] OR "FABS"[All Fields] OR "Tinetti"[All Fields] OR "postural sway"[All Fields] OR "limb stance"[All Fields] OR "cop"[All Fields] OR "centre of pressure"[All Fields] OR "center of pressure"[All Fields]) AND ("Randomized clinical trial"[All Fields] OR "RCT"[All Fields]

OR "random*"[All Fields] OR "Randomized controlled trial"[All Fields] OR "Randomised clinical trial"[All Fields] OR "randomised"[All Fields] OR "Randomised controlled trial"[All Fields])

SCOPUS

TITLE-ABS-KEY("Aged" OR "older" OR "elderly" OR "senior") AND TITLE-ABS-KEY("Virtual Reality" OR "Virtual Reality Exposure Therapy" OR "Exergaming" OR "game-based rehabilitation" OR "head mounted display" OR "commercial video games" OR "video gaming" OR "Nintendo" OR "WiiPD" OR "WII fit" OR "Wii-hab" OR "Xbox" OR "PlayStation" OR "Kinect" OR "VR" OR "Exergames" OR "Virtual Reality Exercise" OR "Active Video Games" OR "Serious games" OR "augmented reality" OR "mixed reality" OR "Telemedicine" OR "Telemedicine" OR "Telehealth" OR "Telerehabilitation" OR "Videoconference" OR "Teletherapy" OR "Teleconference" OR "Telepractice" OR "Teletreatment" OR "Virtual conference" OR "ehealth") AND TITLE-ABS-KEY("postural stability" OR "balance" OR "gait" OR "posture" OR "force plate" OR "tug" OR "up and go" OR "bestest" OR "bbs" OR "berg balance scale" OR "kistler" OR "Dynamic Gait Index" OR "stability" OR "leg stance" OR "SLS" OR "Functional reach test" OR "FRT" OR "Fullerton Advance Balance Scale" OR "FABS" OR "Tinetti" OR "postural sway" OR "limb stance" OR "cop" OR "centre of pressure" OR "center of pressure") AND TITLE-ABS-KEY("Randomized clinical trial" OR "RCT" OR "randomized" OR "Randomized controlled trial" OR "Randomised clinical trial" OR "randomised" OR "Randomised controlled trial") AND NOT TITLE("stroke" OR "parkinson's" OR "parkinson" OR "Chronic Obstructive Pulmonary Disease" OR "COPD" OR "cardiac" OR "pulmonary" OR "neurologic" OR "hemiplegia" OR "paraplegia" OR "neuropathy" OR "arthroplasty" OR "kidney" OR "vestibular loss" OR "amblyopia" OR "fracture" OR "Ménière's" OR "breast cancer" OR "cancer" OR "replacement" OR "Alzheimer's" OR "dementia" OR "down syndrome" OR "low back pain" OR "amputation" OR "Huntington disease" OR "multiple sclerosis" OR "insomnia" OR "Fibromyalgia" OR "cochleovestibular loss" OR "osteoarthritis" OR "osteoporosis" OR "surgery" OR "Diabetes" OR "review" OR "meta-analysis" OR "study protocol" OR "quasi-randomized" OR "Cerebral palsy")

Web of Science

((((ALL=("Aged" OR "older" OR "elder*" OR "senior")) AND ALL=("Virtual Reality" OR "Virtual Reality Exposure Therapy" OR "Exergaming" OR "game-based rehabilitation" OR "head mounted display" OR "commercial video gam*" OR "video gam*" OR "Virtual Reality" OR "Nintendo" OR "WiiPD" OR "WII fit" OR "Wii-hab" OR "Xbox" OR "PlayStation" OR "Kinect" OR "VR" OR "Exergam*" OR "Virtual Reality Exercis*" OR "Active Video Gam*" OR "Serious gam*" OR "augmented reality" OR "mixed reality" OR "Telemedicine" OR "Telemedicine" OR "Telehealth" OR "Telerehabilitation" OR "Videoconferenc*" OR "Teletherapy" OR "Teleconferenc*" OR "Telepractice" OR "Teletreatment*" OR "Virtual conference" OR "ehealth")) AND ALL=("postural stability" OR "balance" OR "gait" OR "posture" OR "force plate" OR "tug" OR "up and go" OR "bestest" OR "bbs" OR "berg balance scale" OR "kistler" OR "Dynamic Gait Index" OR "stability" OR "leg stance" OR "SLS" OR "Functional reach test" OR "FRT" OR "Fullerton Advance Balance Scale" OR "FABS" OR "Tinetti" OR "postural sway" OR "limb stance" OR "cop" OR "centre of pressure" OR "center of pressure")) AND ALL=("Randomized clinical trial" OR "RCT" OR "random*" OR "Randomized controlled trial" OR "Randomized clinical trial" OR "RCT" OR "random*" OR "Randomized controlled trial" OR "Randomised clinical trial" OR "randomised" OR "Randomised controlled trial")) NOT TI=("stroke" OR "parkinson's" OR "parkinson" OR "Chronic Obstructive Pulmonary Disease" OR "COPD" OR "cardiac" OR "pulmonary" OR "neurologic" OR "hemiplegia" OR "paraplegia" OR "neuropathy" OR "arthroplasty" OR "kidney" OR "vestibular loss" OR "amblyopia" OR "fracture" OR "Ménière's" OR "breast cancer" OR "cancer" OR "replacement" OR "Alzheimer's" OR "dementia" OR "down syndrome" OR "low back pain" OR "amputation" OR "Huntington disease" OR "multiple sclerosis" OR "insomnia" OR "Fibromyalgia" OR "cochleovestibular loss" OR "osteoarthritis" OR "osteoporosis" OR "surgery" OR "Diabetes" OR "review" OR "meta-analysis" OR "study protocol" OR "quasi-randomized" OR "Cerebral palsy")

Embase

('aged' OR 'older' OR 'elder*' OR 'senior') NOT 'stroke' NOT 'parkinsons' NOT 'parkinson' NOT 'chronic obstructive pulmonary disease' NOT 'copd' NOT 'cardiac' NOT 'pulmonary' NOT 'neurologic' NOT 'hemiplegia' NOT 'paraplegia' NOT 'neuropathy' NOT 'arthroplasty' NOT 'kidney' NOT 'vestibular loss' NOT 'amblyopia' NOT 'fracture' NOT 'ménières' NOT 'breast cancer' NOT 'cancer' NOT 'replacement' NOT 'alzheimers' NOT 'dementia' NOT 'down syndrome' NOT 'low back pain' NOT 'amputation' NOT 'huntington disease' NOT 'multiple sclerosis' NOT 'insomnia' NOT 'fibromyalgia' NOT 'cochleovestibular loss' NOT 'osteoarthritis' NOT 'osteoporosis' NOT 'surgery' NOT 'diabetes' NOT 'review' NOT 'meta-analysis' NOT 'study protocol' NOT 'quasi-randomized' NOT 'cerebral palsy' AND ('virtual reality exposure therapy' OR 'exergaming' OR 'game-based rehabilitation' OR 'head mounted display' OR 'commercial video gam*' OR 'video gam*' OR 'virtual reality' OR 'nintendo' OR 'wiipd' OR 'wii fit' OR 'wii-hab' OR 'xbox' OR 'playstation' OR 'kinect' OR 'vr' OR 'exergam*' OR 'virtual reality exercis*' OR 'active video gam*' OR 'serious gam*' OR 'augmented reality' OR 'mixed reality' OR 'telemedicine' OR 'telehealth' OR 'telerehabilitation' OR 'videoconferenc*' OR 'teletherapy' OR 'teleconferenc*' OR 'telepractice' OR 'teletreatment*' OR 'virtual conference' OR 'ehealth') AND ('postural stability' OR 'balance' OR 'gait' OR 'posture' OR 'force plate' OR 'tug' OR 'up and go' OR 'bestest' OR 'bbs' OR 'berg balance scale' OR 'kistler' OR 'dynamic

gait index' OR 'stability' OR 'leg stance' OR 'sls' OR 'functional reach test' OR 'frt' OR 'fullerton advance balance scale' OR 'fabs' OR 'tinetti' OR 'postural sway' OR 'limb stance' OR 'cop' OR 'centre of pressure' OR 'center of pressure') AND ('randomized clinical trial' OR 'rct' OR 'random*' OR 'randomized controlled trial' OR 'randomised clinical trial' OR 'randomised' OR 'randomised controlled trial')

Cochrane
"Aged" OR "older" OR "elder*" OR "senior" in All Text NOT "stroke" OR "parkinson's" OR "parkinson" OR "Chronic Obstructive Pulmonary Disease" OR "COPD" OR "cardiac" OR "pulmonary" OR "neurologic" OR "hemiplegia" OR "paraplegia" OR "neuropathy" OR "arthroplasty" OR "kidney" OR "vestibular loss" OR "amblyopia" OR "fracture" OR "Ménière's" OR "breast cancer" OR "cancer" OR "replacement" OR "Alzheimer's" OR "dementia" OR "down syndrome" OR "low back pain" OR "amputation" OR "Huntington disease" OR "multiple sclerosis" OR "insomnia" OR "Fibromyalgia" OR "cochleovestibular loss" OR "osteoarthritis" OR "osteoporosis" OR "surgery" OR "Diabetes" OR "review" OR "meta-analysis" OR "study protocol" OR "quasi-randomized" OR "Cerebral palsy" in Record Title AND "Virtual Reality" OR "Virtual Reality Exposure Therapy" OR "Exergaming" OR "game-based rehabilitation" OR "head mounted display" OR "commercial video gam*" OR "video gam*" OR "Virtual Reality" OR "Nintendo" OR "WiiPD" OR "WII fit" OR "Wii-hab" OR "Xbox" OR "PlayStation" OR "Kinect" OR "VR" OR "Exergam*" OR "Virtual Reality Exercis*" OR "Active Video Gam*" OR "Serious gam*" OR "augmented reality" OR "mixed reality" OR "Telemedicine" OR "Telemedicine" OR "Telehealth" OR "Telerehabilitation" OR "Videoconferenc*" OR "Teletherapy" OR "Teleconferenc*" OR "Telepractice" OR "Teletreatment*" OR "Virtual conference" OR "ehealth" in Title Abstract Keyword AND "postural stability" OR "balance" OR "gait" OR "posture" OR "force plate" OR "tug" OR "up and go" OR "bestest" OR "bbs" OR "berg balance scale" OR "kistler" OR "Dynamic Gait Index" OR "stability" OR "leg stance" OR "SLS" OR "Functional reach test" OR "FRT" OR "Fullerton Advance Balance Scale" OR "FABS" OR "Tinetti" OR "postural sway" OR "limb stance" OR "cop" OR "centre of pressure" OR "center of pressure" in Title Abstract Keyword AND "Randomized clinical trial" OR "RCT" OR "random*" OR "Randomized controlled trial" OR "Randomised clinical trial" OR "randomised" OR "Randomised controlled trial" in Title Abstract Keyword - (Word variations have been searched)

Supplementary References

1. Benitez-Lugo, M.-L., Suárez-Serrano, C., Galvao-Carmona, A., Vazquez-Marrufo, M. & Chamorro-Moriana, G. Effectiveness of feedback-based technology on physical and cognitive abilities in the elderly. *Front. Aging Neurosci.* **14**, (2022).
2. Hou, H.-Y. & Li, H.-J. Effects of exergame and video game training on cognitive and physical function in older adults: A randomized controlled trial. *Appl. Ergon.* **101**, 103690 (2022).
3. Kim, S.-H. & Cho, S.-H. Benefits of Virtual Reality Program and Motor Imagery Training on Balance and Fall Efficacy in Isolated Older Adults: A Randomized Controlled Trial. *Med. Kaunas Lith.* **58**, 1545 (2022).
4. Khanmohammadi, R. *et al.* The effect of video game-based training on postural control during gait initiation in community-dwelling older adults: a randomized controlled trial. *Disabil. Rehabil.* **44**, 5109–5116 (2022).
5. Sonthikul, C., Hadhoh, N., Madeeyoh, N., Ponlakarn, A. & Dolthamsiri, N. Comparative effect of core stabilization exercise and active video gaming on dynamic balance in elderly female people: A single-blind randomized controlled clinical trial. *J. Health Sci. Med. Res.* **40**, 53–65 (2022).
6. Gallardo-Meza, C. *et al.* Effects of 4 Weeks of Active Exergames Training on Muscular Fitness in Elderly Women. *J. Strength Cond. Res.* **36**, 427–432 (2022).
7. Fakhro, M. A., Hadchiti, R. & Awad, B. Effects of Nintendo Wii fit game training on balance among Lebanese older adults. *Aging Clin. Exp. Res.* **32**, 2271–2278 (2020).
8. Morat, M. *et al.* Effects of stepping exergames under stable versus unstable conditions on balance and strength in healthy community-dwelling older adults: A three-armed randomized controlled trial. *Exp. Gerontol.* **127**, 110719 (2019).
9. Lim, J., Cho, J.-J., Kim, J., Kim, Y. & Yoon, B. Design of virtual reality training program for prevention of falling in the elderly: A pilot study on complex versus balance exercises. *Eur. J. Integr. Med.* **15**, 64–67 (2017).
10. Padala, K. P. *et al.* Efficacy of Wii-Fit on Static and Dynamic Balance in Community Dwelling Older Veterans: A Randomized Controlled Pilot Trial. *J. Aging Res.* **2017**, (2017).
11. Kwok, B. C. & Pua, Y. H. Effects of WiiActive exercises on fear of falling and functional outcomes in community-dwelling older adults: a randomised control trial. *Age Ageing* **45**, 621–628 (2016).
12. Schattin, A., Arner, R., Gennaro, F. & de Bruin, E. D. Adaptations of Prefrontal Brain Activity, Executive Functions, and Gait in Healthy Elderly Following Exergame and Balance Training: A Randomized-Controlled Study. *Front. Aging Neurosci.* **8**, 278 (2016).

13. Eggenberger, P., Schumacher, V., Angst, M., Theill, N. & de Bruin, E. D. Does multicomponent physical exercise with simultaneous cognitive training boost cognitive performance in older adults? A 6-month randomized controlled trial with a 1-year follow-up. *Clin. Interv. Aging* **10**, 1335–1349 (2015).
14. Whyatt, C., Merriman, N. A., Young, W. R., Newell, F. N. & Craig, C. A Wii Bit of Fun: A Novel Platform to Deliver Effective Balance Training to Older Adults. *Games Health J.* **4**, 423–433 (2015).
15. Bieryla, K. A. & Dold, N. M. Feasibility of Wii Fit training to improve clinical measures of balance in older adults. *Clin. Interv. Aging* **8**, 775–781 (2013).
16. Jorgensen, M. G., Laessoe, U., Hendriksen, C., Nielsen, O. B. F. & Aagaard, P. Efficacy of Nintendo Wii Training on Mechanical Leg Muscle Function and Postural Balance in Community-Dwelling Older Adults: A Randomized Controlled Trial. *J. Gerontol. Ser. -Biol. Sci. Med. Sci.* **68**, 845–852 (2013).
17. Singh, D. K. A. *et al.* Effects of balance-focused interactive games compared to therapeutic balance classes for older women. *Climacteric* **16**, 141–146 (2013).
18. Franco, J. R., Jacobs, K., Inzerillo, C. & Kluzik, J. The effect of the Nintendo Wii Fit and exercise in improving balance and quality of life in community dwelling elders. *Technol. Health Care* **20**, 95–115 (2012).
19. Pichierri, G., Murer, K. & de Bruin, E. D. A cognitive-motor intervention using a dance video game to enhance foot placement accuracy and gait under dual task conditions in older adults: a randomized controlled trial. *Bmc Geriatr.* **12**, 74 (2012).
20. Pluchino, A., Lee, S. Y., Asfour, S., Roos, B. A. & Signorile, J. F. Pilot Study Comparing Changes in Postural Control After Training Using a Video Game Balance Board Program and 2 Standard Activity-Based Balance Intervention Programs. *Arch. Phys. Med. Rehabil.* **93**, 1138–1146 (2012).
21. Rendon, A. A. *et al.* The effect of virtual reality gaming on dynamic balance in older adults. *Age Ageing* **41**, 549–552 (2012).
22. Toulotte, C., Toursel, C. & Olivier, N. Wii Fit (R) training vs. Adapted Physical Activities: which one is the most appropriate to improve the balance of independent senior subjects? A randomized controlled study. *Clin. Rehabil.* **26**, 827–835 (2012).
23. Alemrany, A. M., Badr, N. M., Farghaly, A. A. & Abed El Ghaffar, H. A. Effect of virtual reality training on risk of falls and quality of life among elderly. *Fizjoterapia Pol.* **21**, 138–143 (2021).
24. Babadi, S. Y. & Daneshmandi, H. Effects of virtual reality versus conventional balance training on balance of the elderly. *Exp. Gerontol.* **153**, 111498 (2021).
25. de Lima, B. E., Passos, G. S., Youngstedt, S. D., Bandeira Santos Junior, L. C. & Santana, M. G. Effects of Xbox Kinect exercise training on sleep quality, anxiety and functional capacity in older adults. *J. Bodyw. Mov. Ther.* **28**, 271–275 (2021).
26. Moreira, N. B., Rodacki, A. L. F., Costa, S. N., Pitta, A. & Bento, P. C. B. Perceptive-Cognitive and Physical Function in Prefrail Older Adults: Exergaming Versus Traditional Multicomponent Training. *Rejuvenation Res.* **24**, 28–36 (2021).
27. Sadeghi, H. *et al.* Effects of 8 Weeks of Balance Training, Virtual Reality Training, and Combined Exercise on Lower Limb Muscle Strength, Balance, and Functional Mobility Among Older Men: A Randomized Controlled Trial. *Sports Health-Multidiscip. Approach* **13**, 606–612 (2021).
28. Chen, P.-J., Penn, I.-W., Wei, S.-H., Chuang, L.-R. & Sung, W.-H. Augmented reality-assisted training with selected Tai-Chi movements improves balance control and increases lower limb muscle strength in older adults: A prospective randomized trial. *J. Exerc. Sci. Fit.* **18**, 142–147 (2020).
29. Yang, C.-M., Chen Hsieh, J., Chen, Y.-C., Yang, S.-Y. & Lin, H.-C. K. Effects of Kinect exergames on balance training among community older adults A randomized controlled trial. *Medicine (Baltimore)* **99**, (2020).
30. Phirom, K., Kamnardsiri, T. & Sungkarat, S. Beneficial Effects of Interactive Physical-Cognitive Game-Based Training on Fall Risk and Cognitive Performance of Older Adults. *Int. J. Environ. Res. Public. Health* **17**, 6079 (2020).
31. Bacha, J. M. R. *et al.* Effects of kinect adventures games versus conventional physical therapy on postural control in elderly people: A randomized controlled trial. *Games Health J.* **7**, 24–36 (2018).
32. Htut, T. Z. C., Hiengkaew, V., Jalayondeja, C. & Vongsirinavarat, M. Effects of physical, virtual reality-based, and brain exercise on physical, cognition, and preference in older persons: a randomized controlled trial. *Eur. Rev. Aging Phys. Act.* **15**, 10 (2018).
33. Ordnung, M., Hoff, M., Kaminski, E., Villringer, A. & Ragert, P. No Overt Effects of a 6-Week Exergame Training on Sensorimotor and Cognitive Function in Older Adults. A Preliminary Investigation. *Front. Hum. Neurosci.* **11**, 160 (2017).
34. Bieryla, K. A. Xbox Kinect training to improve clinical measures of balance in older adults: a pilot study. *Aging*

Clin. Exp. Res. **28**, 451–457 (2016).
35. Chow, D. H. K. & Mann, S. K. F. Effect of Cyber-Golfing on Balance Amongst the Elderly in Hong Kong: A Pilot Randomised Trial. *Hong Kong J. Occup. Ther.* **26**, 9–13 (2015).
36. Gschwind, Y. J. *et al.* The effect of sensor-based exercise at home on functional performance associated with fall risk in older people - a comparison of two exergame interventions. *Eur. Rev. Aging Phys. Act.* **12**, 11 (2015).
37. Karahan, A. Y. *et al.* EFFECTS OF EXERGAMES ON BALANCE, FUNCTIONAL MOBILITY, AND QUALITY OF LIFE OF GERIATRICS VERSUS HOME EXERCISE PROGRAMME: RANDOMIZED CONTROLLED STUDY. *Cent. Eur. J. Public Health* **23**, S14–S18 (2015).
38. Lee, M., Son, J., Kim, J. & Yoon, B. Individualized feedback-based virtual reality exercise improves older women's self-perceived health: A randomized controlled trial. *Arch. Gerontol. Geriatr.* **61**, 154–160 (2015).
39. Sato, K., Kuroki, K., Saiki, S. & Nagatomi, R. Improving Walking, Muscle Strength, and Balance in the Elderly with an Exergame Using Kinect: A Randomized Controlled Trial. *Games Health J.* **4**, 161–167 (2015).
40. Liepa, A., Tang, J., Jaundaldere, I., Dubinina, E. & Larins, V. Feasibility randomized controlled trial of a virtual reality exergame to improve physical and cognitive functioning in older people. *Acta Gymnica* **52**, e2022.007 (2022).
41. Gonçalves, A. *et al.* The Benefits of Custom Exergames for Fitness, Balance, and Health-Related Quality of Life: A Randomized Controlled Trial with Community-Dwelling Older Adults. *Games Health J.* **10**, 245–253 (2021).
42. Lee, K. Virtual Reality Gait Training to Promote Balance and Gait Among Older People: A Randomized Clinical Trial. *Geriatrics* **6**, 1 (2021).
43. Li, X. *et al.* The Impact of a Multitasking-Based Virtual Reality Motion Video Game on the Cognitive and Physical Abilities of Older Adults. *Sustainability* **12**, 9106 (2020).
44. Park, J. & Yim, J. A New Approach to Improve Cognition, Muscle Strength, and Postural Balance in Community-Dwelling Elderly with a 3-D Virtual Reality Kayak Program. *Tohoku J. Exp. Med.* **238**, 1–8 (2016).
45. Gschwind, Y. J. *et al.* ICT-based system to predict and prevent falls (iStoppFalls): results from an international multicenter randomized controlled trial. *Eur. Rev. Aging Phys. Act.* **12**, 10 (2015).
46. Duque, G. *et al.* Effects of balance training using a virtual-reality system in older fallers. *Clin. Interv. Aging* **8**, 257–263 (2013).
47. Bao, T. *et al.* Retention Effects of Long-Term Balance Training with Vibrotactile Sensory Augmentation in Healthy Older Adults. *Sensors* **22**, 3014 (2022).
48. Adcock, M. *et al.* Effects of an In-home Multicomponent Exergame Training on Physical Functions, Cognition, and Brain Volume of Older Adults: A Randomized Controlled Trial. *Front. Med.* **6**, 321 (2020).
49. Bao, T. *et al.* Effects of long-term balance training with vibrotactile sensory augmentation among community-dwelling healthy older adults: a randomized preliminary study. *J. Neuroengineering Rehabil.* **15**, 5 (2018).
50. Schwenk, M. *et al.* Interactive balance training integrating sensor-based visual feedback of movement performance: a pilot study in older adults. *J. Neuroengineering Rehabil.* **11**, 164 (2014).
51. Delbaere, K. *et al.* E-health StandingTall balance exercise for fall prevention in older people: Results of a two year randomised controlled trial. *The BMJ* **373**, (2021).
52. Yerlikaya, T., Oniz, A. & Ozgoren, M. The effect of an interactive tele rehabilitation program on balance in older individuals. *Neurol. Sci. Neurophysiol.* **38**, 180–186 (2021).
53. Wu, G., Keyes, L., Callas, P., Ren, X. & Bookchin, B. Comparison of telecommunication, community, and home-based Tai Chi exercise programs on compliance and effectiveness in elders at risk for falls. *Arch. Phys. Med. Rehabil.* **91**, 849–856 (2010).
54. Lee, K. Home-Based Exergame Program to Improve Physical Function, Fall Efficacy, Depression and Quality of Life in Community-Dwelling Older Adults: A Randomized Controlled Trial. *Healthc. Basel Switz.* **11**, 1109 (2023).
55. Zhao, C. *et al.* The Effects of Active Video Game Exercise Based on Self-Determination Theory on Physical Fitness and Cognitive Function in Older Adults. *J. Clin. Med.* **11**, 3984 (2022).
